# MiR-153 targets the nuclear factor-1 family and protects against teratogenic effects of ethanol exposure in fetal neural stem cells

**DOI:** 10.1242/bio.20147765

**Published:** 2014-07-25

**Authors:** Pai-Chi Tsai, Shameena Bake, Sridevi Balaraman, Jeremy Rawlings, Rhonda R. Holgate, Dustin Dubois, Rajesh C. Miranda

**Affiliations:** Department of Neuroscience and Experimental Therapeutics, College of Medicine, Texas A&M Health Science Center, Bryan, TX 77807-3260, USA

**Keywords:** Fetal alcohol syndrome, Alcohol, Neural stem/progenitor cells, miR-153, Nfia, Nfib, Nicotine, Varenicline

## Abstract

Ethanol exposure during pregnancy is an established cause of birth defects, including neurodevelopmental defects. Most adult neurons are produced during the second trimester-equivalent period. The fetal neural stem cells (NSCs) that generate these neurons are an important but poorly understood target for teratogenesis. A cohort of miRNAs, including miR-153, may serve as mediators of teratogenesis. We previously showed that ethanol decreased, while nicotine increased miR-153 expression in NSCs. To understand the role of miR-153 in the etiology of teratology, we first screened fetal cortical NSCs cultured *ex vivo*, by microarray and quantitative RT-PCR analyses, to identify cell-signaling mRNAs and gene networks as important miR-153 targets. Moreover, miR-153 over-expression prevented neuronal differentiation without altering neuroepithelial cell survival or proliferation. Analysis of 3′UTRs and *in utero* over-expression of pre-miR-153 in fetal mouse brain identified Nfia (nuclear factor-1A) and its paralog, Nfib, as direct targets of miR-153. *In utero* ethanol exposure resulted in a predicted expansion of Nfia and Nfib expression in the fetal telencephalon. In turn, miR-153 over-expression prevented, and partly reversed, the effects of ethanol exposure on miR-153 target transcripts. Varenicline, a partial nicotinic acetylcholine receptor agonist that, like nicotine, induces miR-153 expression, also prevented and reversed the effects of ethanol exposure. These data collectively provide evidence for a role for miR-153 in preventing premature NSC differentiation. Moreover, they provide the first evidence in a preclinical model that direct or pharmacological manipulation of miRNAs have the potential to prevent or even reverse effects of a teratogen like ethanol on fetal development.

## INTRODUCTION

Neural stem cells (NSCs), within the human fetal ventricular zone (VZ), generate most neurons of the adult human brain during a restricted developmental window encompassing the end of the first trimester, through the second trimester of pregnancy (Bystron et al., 2008). In rodent models, this neurogenic-equivalent period encompasses the second half of pregnancy (Noctor et al., 2004; Takahashi et al., 1995). The rate of drug abuse among pregnant women is also highest during these trimesters (SAMHSA, 2009). Therefore, the peak period for maternal consumption of drugs like alcohol, a causative factor in the etiology of the fetal alcohol syndrome (FAS (Jones et al., 1973)), coincides with, and is therefore likely to interfere with the critical period for fetal neurogenesis. In rodent models, maternal ethanol exposure during this neurogenic period has been shown to result in fetal brain growth deficits (Kotkoskie and Norton, 1989; Miller and Nowakowski, 1991; Sudheendran et al., 2013). Importantly, these deficits were not due to NSC death, but rather due to NSC depletion by loss of renewal and premature maturation (Prock and Miranda, 2007; Santillano et al., 2005; Tingling et al., 2013).

A question that arises is “What molecular mechanisms mediate fetal NSC vulnerability and contribute to teratology associated with maternal drug abuse?”. MicroRNAs (miRNAs), a class of small noncoding RNAs that regulate the translation of networks of protein-coding genes, have long been known to control development (Pasquinelli et al., 2000). Therefore, we hypothesized that miRNAs would mediate the effects of teratogens on the growth and maturation of fetal NSCs.

MiR-153, a brain-enriched (Sempere et al., 2004), evolutionarily conserved miRNA located within the *PTPRN2* gene locus, is a candidate mediator of teratogenesis. We identified miR-153 as one of a small cohort of miRNAs that were significantly decreased in fetal NSCs, following ethanol exposure (Sathyan et al., 2007). In that, and subsequent studies (Pappalardo-Carter et al., 2013), we also showed that the suppression of ethanol-sensitive miRNAs individually and collectively, explained some of the teratogenic effects of ethanol. Recently, developmental ethanol exposure was shown to also result in decreased miR-153 in a zebrafish model, and dysregulation of miR-153 in that model in turn resulted in neurobehavioral impairment (Tal et al., 2012). Moreover, nicotine also influenced miR-153 expression (Balaraman et al., 2012), suggesting that miR-153 is a target for other teratogenic agents and drugs of abuse.

In the following series of experiments, we identified cell-signaling gene networks as important targets of miR-153 in fetal cortical NSCs. We further identified the Nfia (nuclear factor-1A) and its paralog, Nfib as direct targets of miR-153, and showed that these and other miR-153 target transcripts were also up-regulated following ethanol exposure. Importantly, we present evidence showing that miR-153 prevents, and even partly reverses the effects of ethanol exposure. Moreover, varenicline, a partial nicotinic acetyl choline receptor (nAChR) agonist (Mihalak et al., 2006), prevented and reversed the effects of ethanol on miR-153 target transcripts. Collectively, these data provide evidence for the efficacy of miRNA-mediated mechanisms in preventing and even reversing effects of teratogen exposure in fetal NSCs.

## RESULTS

**Fig. 1.**
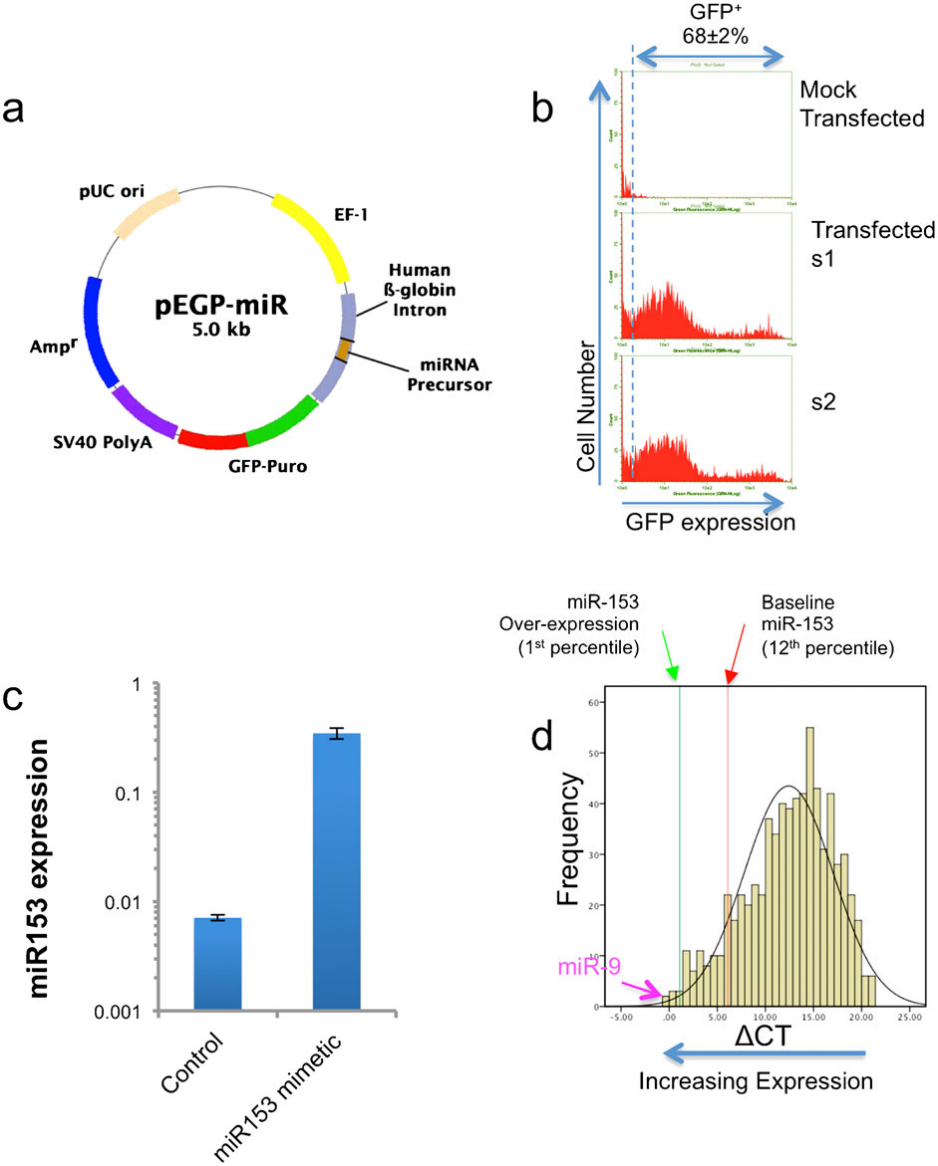
Identification and gene ontology classification of mRNAs that are down-regulated following miR-153 over-expression. (a) Schematic structure of the pre-miR-153/GFP-puromycin expression vector (Cell Biolabs, CA). (b) Sample flow-cytometry frequency histograms documenting transfection efficiency. The upper panel depicts mock-transfected controls, whereas the lower two panels (s1 and s2) show GFP expression following transfection. GFP-transfected cells exhibit a bi-modal distribution, with a mean transfection efficiency of 68±2%. Cells were cultured for 24 hours before labeling with anti-GFP antibody, followed by flow cytometry. (c) Bar graph shows that transfection of neurosphere-derived cells with pre-miR-153 expression vector results in a 30-fold increase in miR-153 expression compared to transfection with vector control. Data from six independent replicates (n = 6) are expressed as mean±SEM. (d) Frequency histogram of miRNA expression (ΔCT relative to U6 snRNA) in neurosphere cultures showing the relative baseline and transfection-induced expression of miR-153. Smaller ΔCT values indicate increased expression. Data show that baseline miR-153 expression is within the upper 12th percentile of all expressed miRNAs, and that over-expression results in a shift to the 1st percentile. However, another ethanol-sensitive miRNA, miR-9 (indicated with arrow), is expressed at a higher baseline level compared to miR-153 over-expression.

To better understand how miRNAs like miR-153 may mediate teratogenesis, we over-expressed miR-153 in fetal NSCs for 24 hours in a transient transfection assay (see Fig. 1a for map of expression construct). Transfection (at an efficiency of ∼68±2%) yielded populations of cells expressing moderate and high levels of GFP (Fig. 1b). Comparative analysis of miR-153 (Exiqon miRCURY qPCR arrays, using previously published protocols (Balaraman et al., 2014)) indicates that miR-153 in untransfected control NSCs is expressed at the upper 12th percentile of all expressed miRNAs. Transfection with the miR-153 expression construct resulted in a ∼32-fold induction of miR-153 compared to the transfection control (t_(10)_ = −8.33, p<8.27e−06, Fig. 1c,d), increasing the relative expression of miR-153 to the upper 1st percentile of all expressed miRNAs (Fig. 1d). At this level of expression, miR-153 is 2.5-fold less abundant than miR-9, another ethanol-sensitive miRNA (Balaraman et al., 2012; Pappalardo-Carter et al., 2013; Sathyan et al., 2007). Therefore, transfection with the miR-153 expression construct resulted in significant over-expression of miR-153, although within the range of miRNA expression observed in NSCs.

**Fig. 2.**
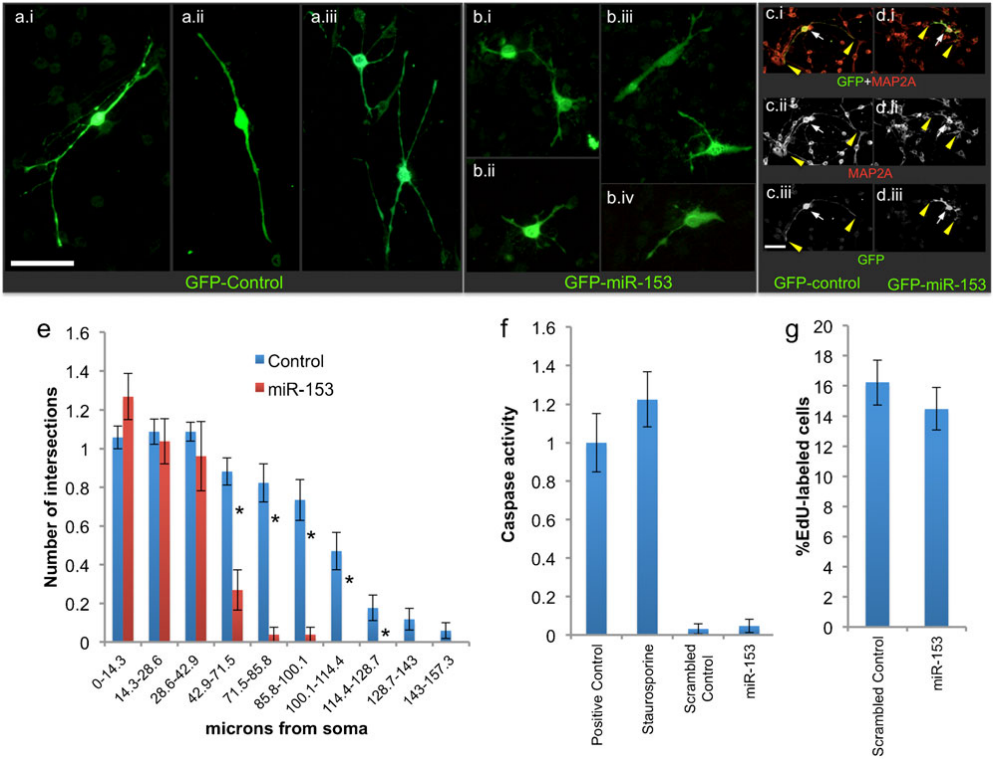
Effects of miR-153 over-expression on differentiation, apoptosis and cell proliferation. (a,b) Photomicrographs depicting GFP immunofluorescent cells cultured under mitogen-withdrawal-induced differentiation conditions on a laminin substrate 48 hours after transfection with GFP control vector (a.i–a.iii) or with GFP-premiR-153 (b.i–b.iv). MiR-153 over-expressing cells exhibited deficient morphological transformation compared to control cells. (c,d) Differentiating control-GFP (c.i–c.iii) and miR-153/GFP (d.i–d.iii) over-expressing cells exhibit co-localized expression of the early neuronal marker Map2a/b (white arrows). Yellow arrowheads show that the GFP label fills the cellular processes and completely overlaps the expression of Map2a/b. Map2a/b immunolabeling also shows deficient morphological transformation following miR-153/GFP over-expression. (e) Bar graph depicts Sholl analysis of neurite length expressed as number of intersections (*y*-axis) as a function of distance from soma (*x*-axis) per cell. MiR-153 over-expressing cells have shorter neurites compared to controls. Data based on analysis of 34 control and 26 miR-153-over-expressing cells. Photomicrographs were obtained from all four quadrants of each culture dish, and cells whose processes showed no overlap with those of an adjacent cell were selected for analysis. Asterisks, all p-values <0.02. (f) Pan-caspase activity was measured as an estimate of apoptosis. Caspase activity was high in U937 cells treated with camptothecin (4 µg/ml for four hours, positive control) and staurosporine (2 µM for two hours)-treated neurosphere cultured cells and low in neurosphere cultures transfected with control or pre-miR-153 expressing vectors. There was no statistically significant difference between the latter two conditions (n = 6). (g) EdU incorporation into DNA was used as a marker for cell proliferation. The percentage of labeled cells was not significantly different indicating that cell proliferation was not altered by miR-153 overexposure. Data based on three independent transfection experiments. One photomicrograph was obtained from each quadrant of the culture well (three culture wells per sample) and labeled and total number of cells counted. Scale bars: 100 µm.

Following transfection with control-GFP or miR-153-GFP expression vectors, neurosphere-derived cells were cultured for an additional 48 hours on a laminin substrate, in a mitogen-withdrawal-induced differentiation paradigm that has previously been shown to result in transformation of NSCs into early migratory neurons (Camarillo et al., 2007; Camarillo and Miranda, 2008; Tingling et al., 2013). Control NSCs exhibited morphological transformation into bipolar and multipolar cells with elongated processes (Fig. 2a.i–a.iii), whereas miR-153-GFP transfected cells exhibited deficient morphological transformation (Fig. 2b.i–b.iv). However, both control-GFP and miR-153-GFP over-expressing cells expressed immunofluorescence for MAP2a/b (Fig. 2c.i–c.iii vs Fig. 2d.i–d.iii), an early-appearing neuronal marker (Papandrikopoulou et al., 1989), indicating that miR-153 over-expressing cells retain at least some aspects of neuronal lineage commitment, despite reduced neurite extension. It is possible that longer periods of miR-153 over-expression may result in a more profound suppression of differentiation. In strongly GFP-labeled cells, GFP fluorescence completely overlapped with MAP2a/b immuno-label, and therefore neurite outgrowth was assessed in cells that expressed strong GFP label (Fig. 2d). Sholl analysis (Fig. 2e) indicated that differentiating cells over-expressing GFP-miR-153 had significantly shorter neurites compared to cells transfected with the control GFP plasmid (Multivariate Analysis of Variance–Pillai's Trace Statistic (MANOVA_PTS_), F_(10,49)_ = 5.11, p<4.35E−05). However, miR-153 over-expression did not result in significant apoptosis (Fig. 2f) or change in cell proliferation (Fig. 2g).

### Identification of miR-153-regulated candidate genes

**Fig. 3.**
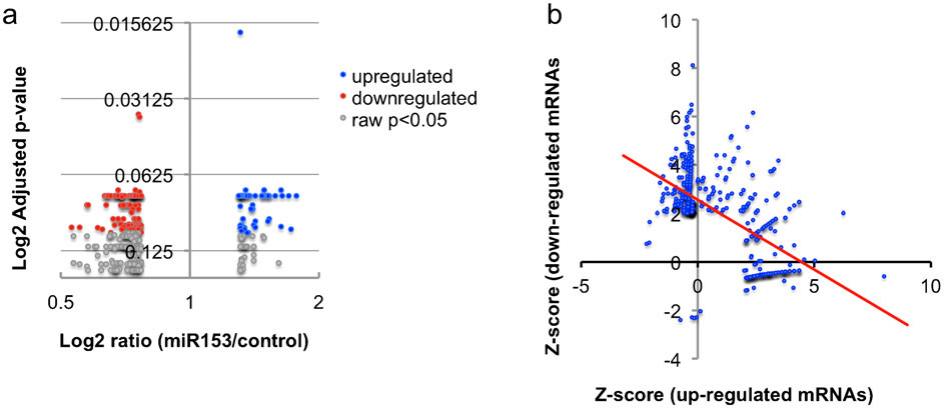
Microarray analysis of gene expression following miR-153 over-expression. (a) Volcano-plot illustrates relationship between log_2_(mRNA expression ratio) and log_2_(FDR-corrected p-value) in miR-153 over-expressing cultures compared to controls. Filled red and blue circles indicate mRNA transcripts that are suppressed or induced, respectively, by more than 1.3-fold following miR-153 over-expression, at an FDR (Benjamini and Hochberg)-adjusted p<0.1. Gray circles indicate genes that reached statistical significance at a raw p<0.05. Data summarized from 6 independent replicate experiments. (b) The plot shows the correlation of gene ontology analysis of mRNA transcripts that were suppressed and induced following miR-153 over-expression. The *x*-axis depicts the z-score of ontology analysis of mRNAs induced by miR-153. The *y*-axis indicates the z-score of ontology analysis of mRNAs suppressed by miR-153. The red line is the regression line that indicates a negative linear relationship between ontology classes of miR-153 suppressed and induced mRNA transcripts.

To assess the mechanisms underlying miR-153 mediated suppression of differentiation we performed microarray analysis to first identify miR-153 targets in fetal NSCs. Transcript profiles were assessed from RNA samples obtained 24 hours following transfection with GFP/miR-153 or GFP control vectors (6 independent replicates in each condition, microarray data submitted to NCBI-GEO (accession number GSE49684)). Data analysis showed that miR-153 over-expression resulted in statistically significant down-regulation of 133 genes (at a false discovery rate α = 0.1, of a total of 860 transcripts down-regulated by ≥1.3-fold, Fig. 3a; Table 1). Ontology analysis showed that there was a negative correlation between ontologies associated with down-regulated and up-regulated transcripts (Pearson's r = −0.592, p<0.1E−10, Fig. 3b), indicating that the functions of the down-regulated, including presumably conventionally 3′UTR-targeted mRNAs, did not overlap with the functions of the up-regulated genes. Transcripts that were down-regulated following miR-153 over-expression were significantly overrepresented in ontological categories related to synaptic transmission and G-protein coupled receptor (GPCR) signaling (Table 2). Moreover, pathway analysis (Kyoto Encyclopedia of Genes and Genomes, KEGG) identified “Neuroactive ligand–receptor interaction” as a significantly suppressed pathway. These bioinformatics analyses suggest that in NSCs, miR-153 suppresses pathways that are important for the function of differentiated neurons.

**Table 1.**
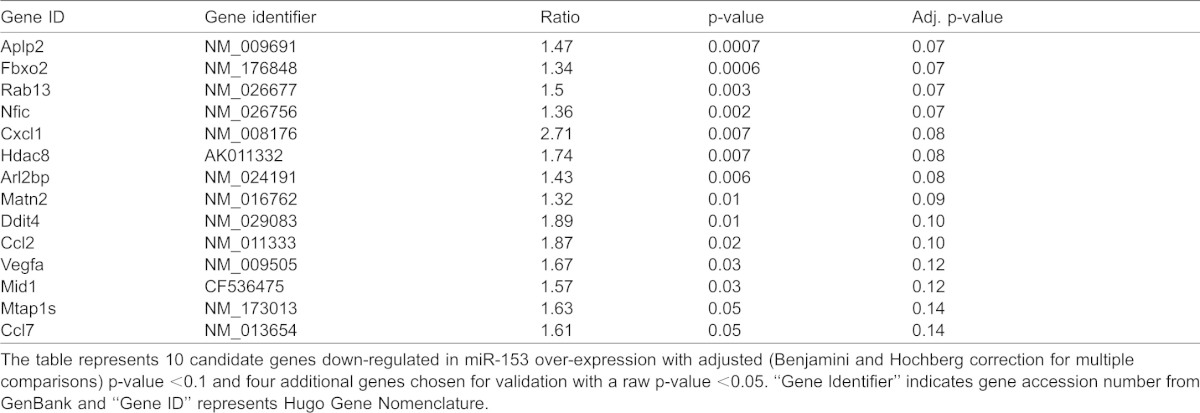
Sample miR-153 candidate gene targets from microarray analysis

**Table 2.**
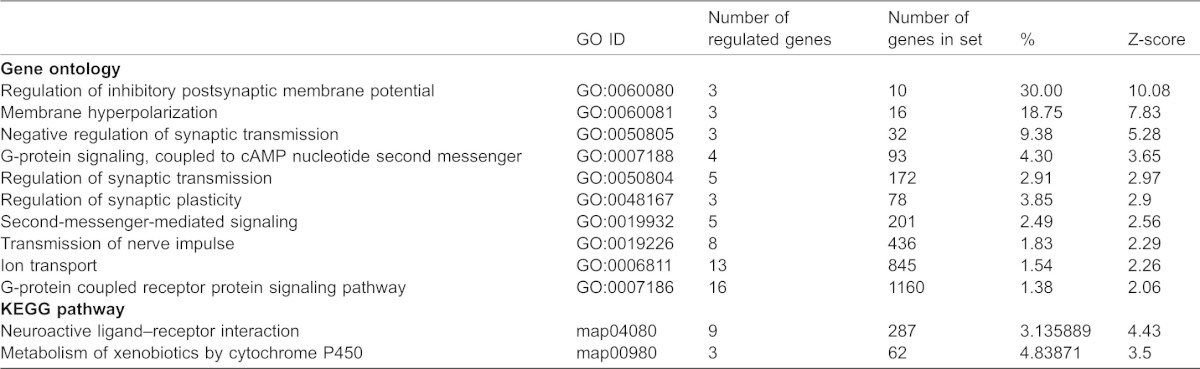
List of major gene ontologies (GO) and KEGG pathways down-regulated by miR-153 with a Z-score >2.0 (indicating that this ontology is over-represented among the regulated genes compared to 95% of all other possible ontologies and pathways)

Interestingly, miRNAs are also known to induce gene expression by binding to alternate target sites outside 3′UTRs, including promoter regions (Place et al., 2008). *In silico* analysis (MirWalk, (Dweep et al., 2011)) of the top six statistically significantly induced RNA transcripts, Patl2, Dhrs13, Rps25, Rai14, Foxo3, and EG665934/GM7854 (FDR-corrected α = 0.02), predicted that two, Rai14 and Rps25, each contained a miR-153 target site with a seed region of 9 nt in length (p<0.037) within the presumptive promoter, a 2 kb upstream gene flanking region. The sixth target, EG665934/GM7854, a candidate long noncoding RNA (lncRNA), is predicted (RNAhybrid, (Krüger and Rehmsmeier, 2006)) to contain a miR-153 target site with a 10 nt seed region. The possibility that these are functional sites remains to be determined. However, these data do suggest the possibility that some of the induced transcripts may be direct targets of miR-153 as well.

**Fig. 4.**
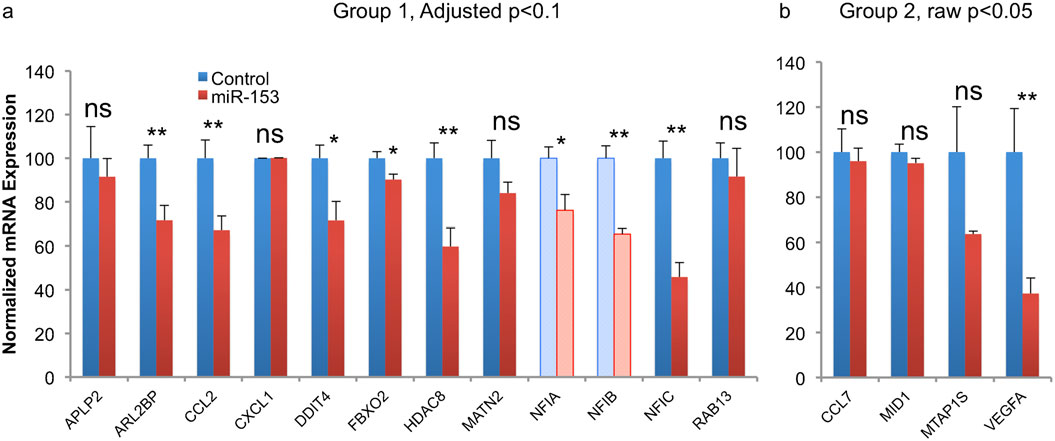
RT-PCR validation of candidate miR-153-regulated mRNAs. Bar graphs depict the real time RT-PCR quantification of mRNAs in vector control and miR-153 over-expression conditions for candidate mRNA transcripts identified from the microarray experiment that achieved the adjusted p-value cut-off of 0.1 (a) and raw p-value of 0.05 (b), respectively. The *y*-axis indicates normalized mRNA expression (expressed as 1/2ΔCt relative to 18s RNA). Data were expressed as mean±SEM and quantified from 6 independent replicates (*p<0.001; **p<0.05).

Nineteen genes were selected for further assessment. These included 10 candidate miR-153-repressed transcripts that reached an FDR-corrected p<0.1 (Aplp2, Arl2bp, Ccl2, Cxcl1, Ddit4, Fbxo2, Hdac8, Matn2, Nfic, Rab13), and 4 with an uncorrected p<0.05 (Ccl7, Mid1, Mtap1s, Vegfa). Three genes, Akt1, Foxj2, and Mkln, were chosen as validation controls that were not statistically significantly altered by miR-153 over-expression. Additionally, because the microarray screen identified Nfic as a candidate target for miR-153, we also assessed other members of the nuclear factor family, Nfia and Nfib. The Nfia family is a particularly important target because of their implicated role in suppressing NSC self-renewal (Namihira et al., 2009; Piper et al., 2010), promoting gliogenesis (Barry et al., 2008; Deneen et al., 2006), and neuronal differentiation and migration (Wang et al., 2010). Moreover, Nfia haplo-insufficiency is associated with brain malformations including agenesis of the corpus callosum, ventriculomegaly, and Chiari-type-1 malformations (Lu et al., 2007), features associated with fetal alcohol exposure. None of the validation control mRNAs were altered following miR-153 over-expression. Among the genes that met the adjusted-p<0.1 criteria, Arl2bp (t_(10)_ = 3.09, p<0.01), Ccl2 (t_(10)_ = 3.14, p<0.01), Ddit4 (t_(10)_ = 2.70, p<0.02), Fbxo2 (t_(10)_ = 2.48, p<0.03), Hdac8 (t_(10)_ = 3.66, p<0.004) and importantly, Nfia, Nfib and Nfic (t_(10)_ = 2.67, p<0.024; t_(10)_ = 5.49, p<0.001; t_(10)_ = 5.28, p<0.001, respectively, Fig. 4a), were significantly decreased following miR-153 over-expression in the validation experiment. Among the cohort of genes that exceeded the ‘raw’ p<0.05 criterion, only Vegfa (t_(10)_ = 3.07, p<0.012, Fig. 4b) was significantly reduced following miR-153 exposure in the validation sample.

### Identification of 3′UTR regulatory motifs in miR-153 targeted transcripts

mRNAs that were down-regulated following miR-153 over-expression may include both direct and indirect targets. Moreover, miR-153 over-expression may block NSC maturation, thereby indirectly uncovering gene expression differences in the microarray analyses. To directly address these possibilities, we first utilized bioinformatic tools (Targetscan, http://www.targetscan.org) to perform an *in silico* analysis 3′UTRs. Nfia and Nfib each contained several predicted miR-153 binding sites in their 3′UTRs with high aggregate P_CT_s (probability of conserved targeting (Friedman et al., 2009)) of 0.99 and 0.96, respectively, indicating that miR-153 interactions with Nfia/Nfib 3′UTRs are likely to be evolutionarily conserved. Matn2 and Vegfa 3′UTRs were assessed as controls. Matn2 was not validated as a miR-153 target in our earlier miR-153 over-expression study, scored a lower P_CT_ of 0.78, and is predicted to contain a 3′UTR miR-153 target site in primates, but not rodents. Vegfa 3′UTR contained no predicted miR-153 binding sites, although VEGFa mRNA was significantly reduced following miR-153 transfection. Moreover, Vegfa is the most highly expressed cytokine in proliferating NSCs, and its expression levels decrease significantly with neural differentiation (Camarillo et al., 2007), suggesting that this cytokine is important for NSCs.

**Fig. 5.**
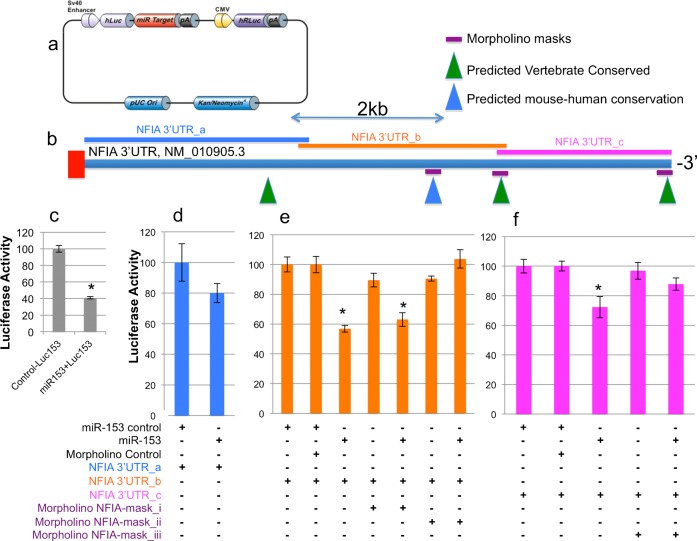
Identification of Nfia 3′UTR as a direct target of miR-153. (a) Schematic structure of the luciferase reporter constructs containing murine Nfia 3′UTR fragments (GeneCopoeia, Rockville, MD). (b) Schematic of the full length of Nfia 3′UTR depicting the location of the three Nfia 3′UTR fragments (Nfia 3′UTR_a in blue, Nfia 3′UTR_b in orange, Nfia 3′UTR_c in pink) that were cloned into luciferase reporters for studies. Green triangles indicate the predicted miR-153 binding sites that are conserved among vertebrates, whereas the blue triangle illustrates the predicted miR-153 binding site that shares conservation between mouse and human. Purple bars represent the morpholinos used to protect the predicted miR-153 binding sites. (c) We assayed firefly activity relative to RLuc luciferase activity in NSCs 24 h after transfection with the Luc_miR153 binding site reporter construct as the positive control and transfected control or miR-153 mimetics. Bar graphs represent luciferase activity normalized to the mean activity of samples transfected with the miR-153 control vector. The *x*-axis depicts treatment conditions. The *y*-axis indicates normalized luciferase activity. (d–f) NSCs were transfected with luciferase reporter constructs containing different fragments of Nfia 3′UTR, (d) 3′UTR_a, (e) 3′UTR_b, (f) 3′UTR_c, with control or miR-153 mimetics for 24 hours. Additional control or antisense morpholinos, (e) mask_i, mask_ii, and (f) mask_iii, used to protect the miR-153 binding sites were co-transfected along with other constructs as indicated on the *x*-axis. Bars are normalized to the relative firefly units of samples treated with the transfected control. Data were expressed as mean±SEM (n = 5).

**Fig. 6.**
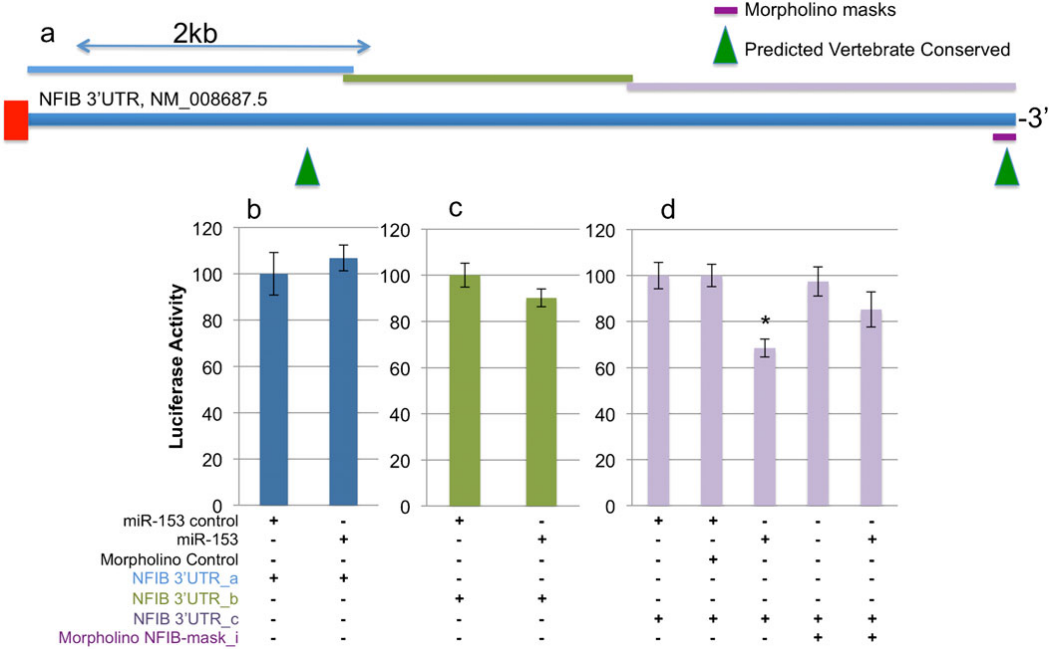
Nfib is a direct target of miR-153. (a) Schematic shows the full length Nfib 3′UTR in relation to the three 3′UTR fragments (Nfib 3′UTR_a in blue, 3′UTR_b in green, 3′UTR_c in purple) that were cloned into luciferase constructs for studies. Green triangles indicate the predicted miR-153 binding sites that are conserved among vertebrates. Purple bars represent the morpholinos used to mask the miR-153 binding sites. (b–d) Firefly activity relative to RLuc luciferase activity is determined in NSCs 24 h after transfection with luciferase reporter constructs containing different fragments of Nfib 3′UTR, (b) 3′UTR_a, (c) 3′UTR_b, (d) 3′UTR_c, with control or miR-153 mimetics. Additional control or antisense morpholinos, (d) Nfib-mask_i, used to protect the predicted miR-153 binding sites in Nfib 3′UTR were co-transfected into same samples as indicated. Data were normalized to the samples treated with the transfected control. The *x*-axis depicts treatment conditions. The *y*-axis indicates normalized luciferase activity. Data were expressed as mean±SEM (n = 5).

Luciferase-reporter constructs (Fig. 5a) containing murine 3′UTRs of Nfia, Nfib, the negative controls, Matn2 and Vegfa, and as a positive control, Luc153, were each transfected into single cell suspensions derived from neurosphere cultures. Some cell aliquots were also co-transfected with pre-miR-153 or control expression vectors. Additionally, antisense morpholino oligonucleotides can be used to protect 3′UTR target sites from miRNAs, by interfering with miRNA-regulated translation (Choi et al., 2007). We therefore co-transfected either scrambled (control), or antisense morpholino oligonucleotides (Table 3; Fig. 5b, Fig. 6a) into some samples, to mask predicted miR-153 binding sites. At the end of 24 hours, luciferase activity, normalized to Renilla luciferase, was determined. Because of their length, the 3′UTR region within the Nfia and Nfib transcripts were each fragmented into three parts and cloned into separate constructs downstream from the luciferase reporter (Fig. 5b, Fig. 6a).

**Table 3.**
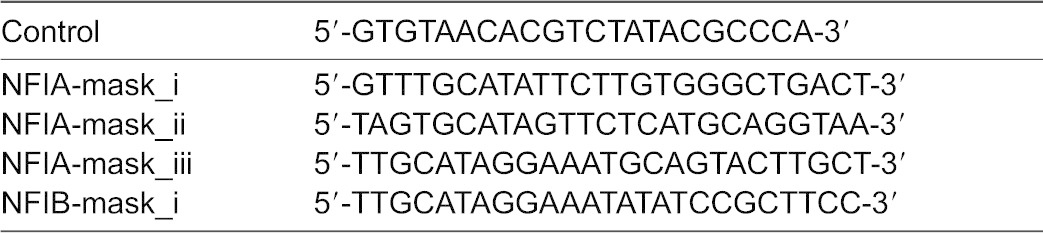
Sequences of morpholino oligonucleotides

MiR-153 over-expression resulted in a 60% reduction in luciferase activity from the co-transfected luciferase expression construct with miR-153 binding sites in the 3′UTR (Luc153, Fig. 5c, p<6.64E−07). In the case of Nfia, miR-153 targeting of Luc-Nfia-3′UTR_a did not significantly repress luciferase activity (Fig. 5d). However, miR-153 did repress luciferase activity from the Luc-Nfia-3′UTR_b and from the Luc-Nfia-3′UTR_c constructs (overall ANOVAs, F_(6,28)_ = 17.23, p<3.07E−08, and F_(4,18)_ = 4.59, p<0.01, respectively, Fig. 5e,f). Masking morpholinos, Nfia_mask_ii and Nfia_mask_iii, but not Nfia_mask_i, were able to completely protect against miR-153-mediated translation repression. Collectively, these data indicate that two out of four predicted miR-153 binding sites localized near the end of the Nfia-3′UTR (Nfia/NM_010905.3_7285–7306_ and Nfia/NM_010905.3_9451–9469_) mediated miR-153 translation repression in fetal neuroepithelial cells. Similarly, Nfib was a direct target of miR-153. Translation of Luc-Nfib-3′UTR_a (Fig. 6b) or Luc-Nfib-3′UTR_b (Fig. 6c) was not repressed by miR-153 over-expression. However, translation of Luc-Nfib-3′UTR_c (Fig. 6d) was repressed by miR-153 over-expression (overall ANOVA, F_(4,18)_ = 4.6, p<0.01). The Nfib-mask_i morpholino protected the second, more distally located predicted miR-153 binding site (Nfib/NM_008687.5_6559–6566_), located near the 3′-end of the 3′UTR, and prevented translation repression.

**Fig. 7.**
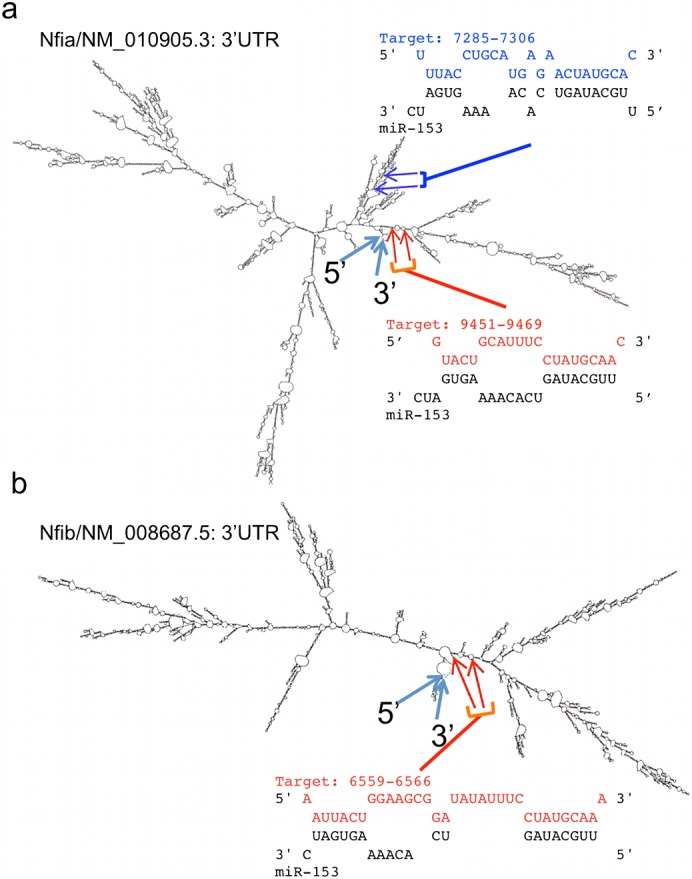
*In silico* analysis of the RNA folding structure of Nfia and Nfib. Predicted secondary structure conformation of the 3′UTR of (a) Nfia and (b) Nfib. Locations of the 5′ and 3′ends of the 3′UTR sequences are marked with blue arrows. (a) Two miR-153 binding sites, target_7285 and target_9451, validated from luciferase assay above are shown on Nfia 3′UTR. Target_7285 is located close to complex stem–loop structures, while target_9451 localizes to a linear portion, of the Nfia 3′UTR. MiR-153 sequences are shown in black while the matching binding site sequences are illustrated in blue (target_7285) and red (target_9451). (b) One miR-153 binding site, target_6559, validated from luciferase analysis is located on the linear portion of Nfib 3′UTR and is labeled in red, whereas miR-153 sequence is shown in black.

**Fig. 8.**
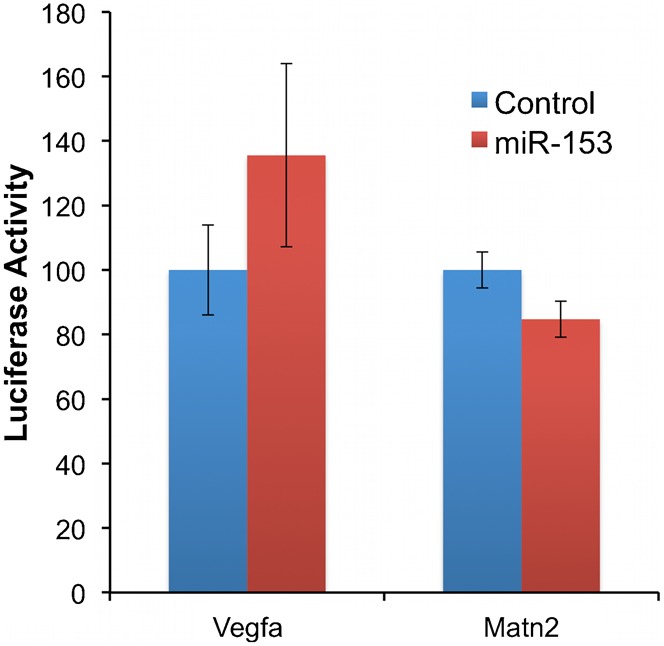
Matn2 and Vegfa are not direct targets of miR-153. Firefly activity relative to RLuc luciferase activity was measured in NSCs 24 hours after transfection with control or miR-153 mimetics and the luciferase construct containing 3′UTR of Matn2 or Vegfa. Bars are normalized to the relative firefly units of samples treated with the transfected control. The *x*-axis depicts treatment conditions (control or miR-153). The *y*-axis indicates normalized luciferase activity. Data were expressed as mean ± SEM (n = 5).

*In silico* analysis (Mfold, http://mfold.rna.albany.edu (Zuker, 2003)) of the folding of the Nfia 3′UTR indicates that miR-153 target site Nfia/NM_010905.3_77285–7306_ localizes to a complex of branched stem–loop structures, whereas Nfia/NM_010905.3_9451–9469_ localized to a predicted linearized portion of the 3′UTR. However, both sites are positioned proximate to the 3′ and 5′ termini of the 3′UTR (Fig. 7a). Analysis of Nfib-3′UTR folding showed that the Nfib/NM_08689.5_6559–6566_ site, like Nfia/NM_010905.3_9451–9469_, was located within a region predicted to assume a more linearized structure, in close physical proximity to the 5′ and 3′-ends of the 3′UTR (Fig. 7b). These data indicate that miR-153 binding sites within Nfia and Nfib 3′UTRs are positioned to influence translation activity within the open reading frame (ORF). Neither Matn2 nor Vegfa 3′UTRs exhibited evidence for regulation by miR-153 (Fig. 8), indicating that these were not direct miR-153 targets in NSCs.

### MiR-153 modulates Nfia and Nfib expression in mouse fetal brains

**Fig. 9.**
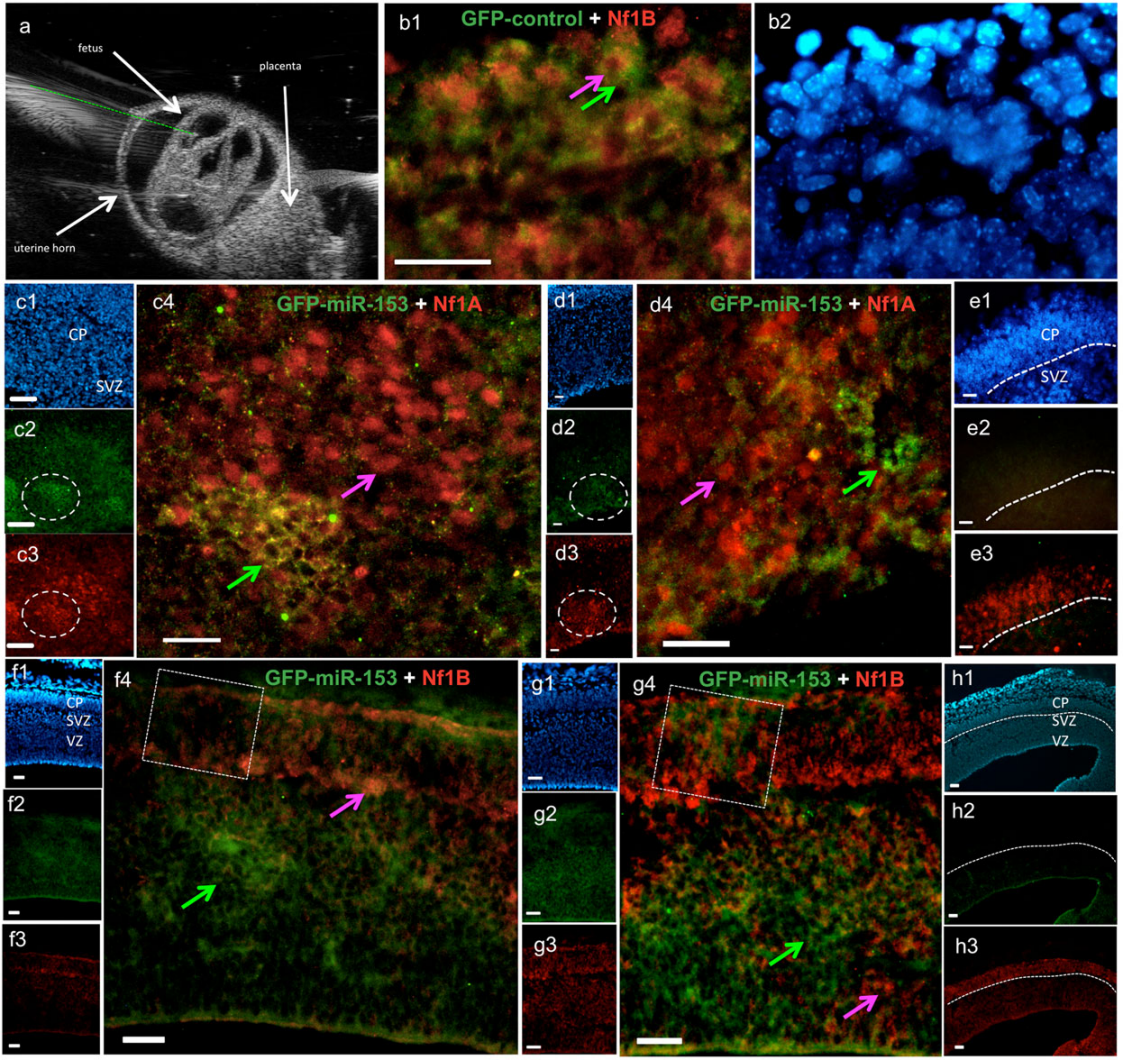
MiR-153 regulates Nfia and Nfib expression in fetal brains. (a) Photo-micrograph depicts ultrasound-guided trans-uterine insertion of a micro-capillary pipette (dashed green line) into the lateral ventricle in a GD13 fetal brain. Following *in utero* electroporation of control-GFP or miR-153/GFP vectors, fetuses were maintained for an additional period of 48 hours, before being analyzed at GD15.5. Double immunohistochemistry of anti-GFP with anti-Nfia or anti-Nfib in control-GFP (b) or Pre-miR-153-GFP (c–h) transfected GD15.5 mouse frozen sections. (b1,b2) Photomicrograph shows (b1) control-GFP (green) localizes to the cytoplasm of nuclear Nfib-labeled (red) neurons of the cortical plate and (b2) DAPI-counterstained nuclei. (c–h) Panels c1–h1, c2–h2 and c3–h3 show low magnification images of the same sections counterstained with DAPI (c1–h1) to visualize nuclei, or immuno-fluorescently labeled for GFP (c2–h2) as a marker for miR-153 over-expression, or Nfia (c3–e3) and Nfib (f3–h3). (c4,d4,f4,g4) High magnification photomicrographs showing that GFP expression from the pre-miR-153/GFP construct does not co-localize with nuclear immuno-labeling for Nfia (c4,d4) or Nfib (f4,g4). Residual cytoplasmic labeling in GFP/miR-153 over-expressing cells, represented by yellow immunofluorescence (e.g. c4), may represent incompletely suppressed translation or residual immuno-reactivity due to products of stalled translation. Dotted circles indicate regions depicted in high magnification images. Dotted squares depict regions of cortical plate with disrupted expression of Nfib overlying strong GFP expression in the ventricular/sub-ventricular zones. Pink arrows show nuclei immuno-stained for Nfia or Nfib, while green arrows indicate strong cytoplasmic miR-153-GFP immuno-staining. VZ: ventricular zone; SVZ: subventricular zone; CP: cortical plate. Scale bars: 25 µm.

Evidence for translational regulation by miR-153 acting at 3′UTRs suggested that Nfia and Nfib were direct miR-153 targets. We therefore further tested this association in an *in vivo* model. Pre-miR-153-GFP or control-GFP expression constructs were delivered to the telencephalic wall of GD13.5 fetuses by intrauterine injection under ultrasound guidance (Fig. 9a) followed by electroporation. After 48 hours (GD15.5), fetuses were euthanized, fixed, and cryo-sectioned. The presence of anti-GFP immunofluorescence was used as a localization marker for cellular over-expression of miR-153. Following *in utero* electroporation with the control-GFP vector, GFP-immuno-reactivity was localized to the cytoplasm of cortical cells that also expressed nuclear Nfia and Nfib (e.g. Fig. 9b–d) immuno-reactivity, indicating that the expression vector did not non-specifically interfere with target gene expression. In contrast, following *in utero* electroporation of the pre-miR-153/GFP construct, GFP immunopositive cells showed little-to-no immunofluorescence for either Nfia or Nfib, whereas adjacent GFP-negative cells exhibited strong nuclear localization of Nfia and Nfib immunoreactivity (Fig. 9c1–c4,d1–d4,f1–f4,g1–g4). In adjacent regions of brain tissue that exhibited little-to-no GFP immunofluorescence, the expression and laminar organization of Nfia and Nfib were undisturbed (Fig. 9e1–e3,h1–h3). However, in regions where there was strong GFP immunofluorescence localized within the ventricular and sub-ventricular zones, expression of Nfia and Nfib in the overlying cortical plate was also disrupted (e.g. Fig. 9f4), although GFP itself was not localized to the cortical plate. Finally, expression of miR-153/GFP in groups of ventricular zone cells appeared to result in compensatory Nfia and Nfib up-regulation in adjacent GFP-negative cells (e.g. Fig. 9d4,g4, green vs purple arrows), suggesting the presence of adaptive communication mechanisms between adjacent neural progenitors. These data show that *in vivo* over-expression of miR-153 results in disrupted translation from target Nfia and Nfib mRNA transcripts.

HDAC8 lacks a predicted miR-153 target site within its 3′UTR, but we examined its expression following miR-153 over-expression because it is the earliest type-1 HDAC to be expressed during neurogenesis in the fetal murine telencephalon (Murko et al., 2010) and is implicated in the etiology of the Wilson–Turner X-linked (Harakalova et al., 2012) and Cornelia de Lange (Deardorff et al., 2012) syndromes, both of which are characterized by cognitive impairment. HDAC8-immunoreactivity was localized predominantly to the cytoplasm of cells within the VZ and SVZ, consistent with previously observed cytoplasmic localization in neural (Takase et al., 2013) and non-neural (Waltregny et al., 2004) tissues. Although its mRNA transcript was suppressed in neurosphere cultures following miR-153 over-expression (Fig. 3), *in vivo* expression of HDAC8-immunoreactivity was not altered by miR-153 over-expression (data not shown). This discrepancy between mRNA and protein expression is consistent with recent literature that suggests that class I HDAC family members are stabilized by post-translational modification (Citro et al., 2013).

**Fig. 10.**
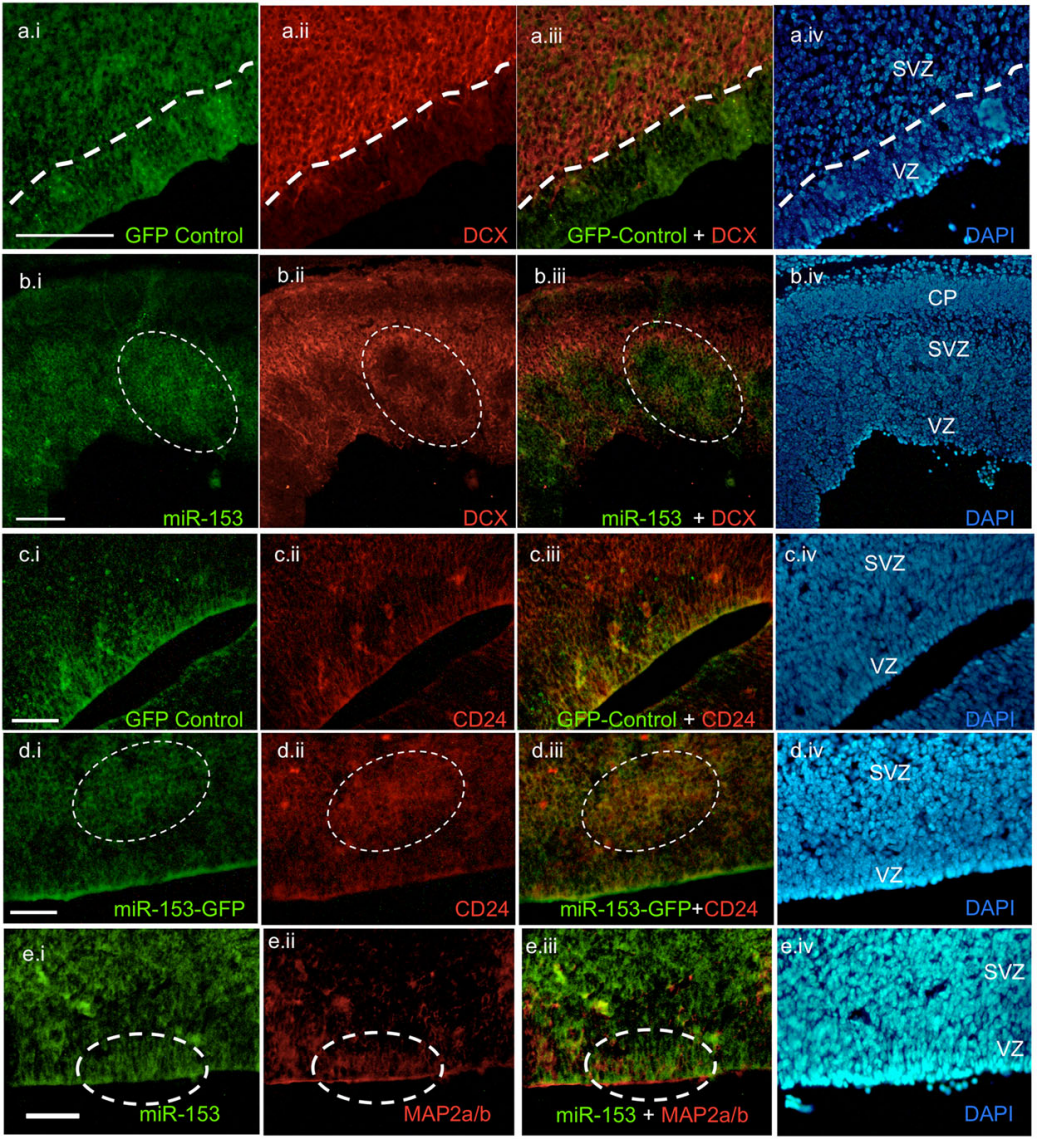
Relationship between miR-153 over-expression and the expression of the neuronal differentiation marker DCX, and the neuronal lineage stem cell marker, CD24 and Map2A, in the cerebral cortical VZ and SVZ. In each row, panel ‘i’ depicts miR-153-GFP or control GFP expression, panel ‘ii’ depicts immunofluorescence for DCX, CD24 or Map2A, panel ‘iii’ depicts combined immunofluorescence and panel ‘iv’ depicts DAPI labeling of cell nuclei. (a,b) DCX-immunofluorescence is localized to the SVZ, but not VZ (b.ii,c.ii). Control-GFP over-expression (a.i–a.iv) does not alter DCX expression in the SVZ; however, miR-153 over-expression (b.i–b.iv, circled areas) results in loss of DCX expression in the SVZ. (c–d) CD24-immunofluorescence localizes to VZ and SVZ in GFP-control (c.i–c.iv) and following miR-153 over-expression (d.i–d.iv). miR-153 over-expression does not result in a loss of CD24 immunofluorescence (white circles). (e.i–e.iv) MiR-153 over-expression does not result in a loss of MAP2a/b expression in newly generated neurons of the VZ (white circles). VZ: ventricular zone; SVZ: subventricular zone; CP: cortical plate. Scale bars: 50 µm.

To further assess the role of miR-153 in neural differentiation, we examined the expression of a marker for early migrating and differentiating neurons (des Portes et al., 1998), doublecortin (DCX), which is not predicted to contain a miR-153 binding site in its 3′UTR, and is therefore unlikely to be a direct miR-153 target. Over-expression of miR-153/GFP within the SVZ coincided with loss of expression of DCX-like immunofluorescence (Fig. 10b.i–b.iv vs Fig. 10a.i–a.iv), indicating that miR-153 over-expression results in a loss of neuronal differentiation, consistent with gene ontology analyses reported above, which indicated that miR-153 over-expression resulted in repression of differentiation-related mRNAs. We previously showed that ethanol exposure resulted in decreased expression of CD24, the neuronal lineage commitment marker, both *in vivo*, and in neurosphere cultures (Tingling et al., 2013). However, miR-153 over-expression did not result in altered expression of CD24-immunofluorescence (Fig. 10d.i–d.iv vs Fig. 10c.i–c.iv), indicating no effect on this ethanol-sensitive marker for neuronal-lineage committed neural precursors. Moreover, miR-153 over-expression also did not result in loss of MAP2a/b immunofluorescence in newly differentiating neurons of the VZ (Fig. 10e.i–e.iv), consistent with the lack of *ex vivo* effects of miR-153 on MAP2a/b expression in neurosphere cultures (Fig. 2c,d).

### Ethanol disrupts the expression pattern of Nfia and Nfib in mouse fetal brains

**Fig. 11.**
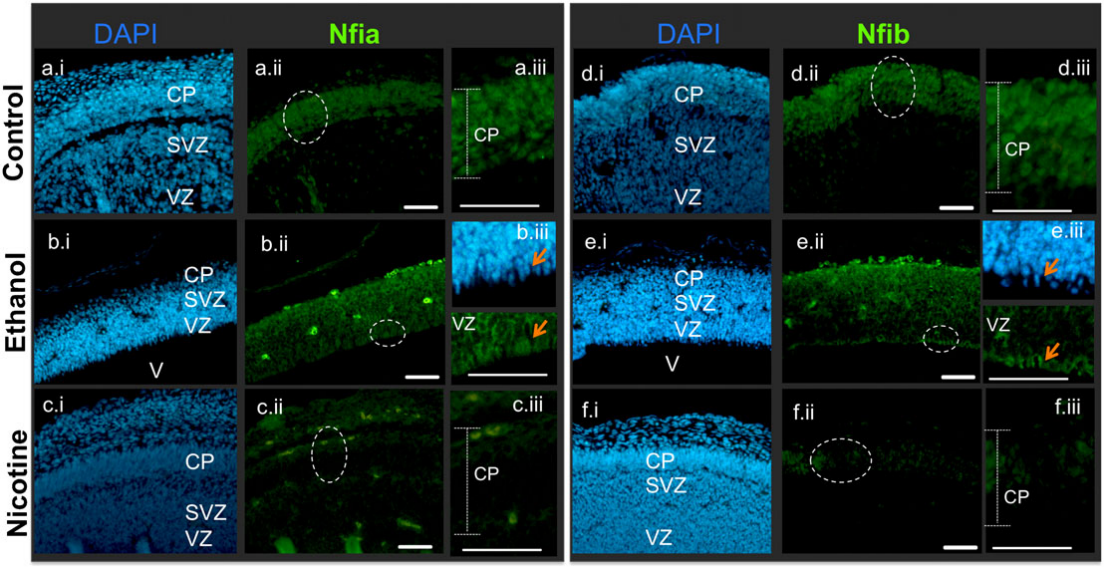
*In utero* ethanol exposure results in expanded Nfia and Nfib immuno-labeling in fetal brains while nicotine exposure results in decreased Nfia and Nfib expression. DAPI-stained sections (a.i–f.i) and their corresponding immuno-fluorescence for Nfia (a.ii–c.ii, and higher magnification inset, a.iii–c.iii) or Nfib (d.ii–f.ii, and higher magnification inset, d.iii–f.iii) in tissue sections obtained from control (upper panel, a,d), ethanol-exposed (middle panel, b,e), and nicotine-exposed (lower panel, c,f) fetal brains. Immuno-label for Nfia and Nfib are mainly localized in the developing cortical plate in control animals, but is spread through the VZ and SVZ in ethanol-exposed animals. White circles in panel ‘ii’ identify regions depicted in panel ‘iii’. Orange arrows identify Nfia and Nfib immuno-reactivity in mitotically active neuroepithelial cells within the apical region of the VZ, following ethanol exposure. Data obtained from four fetuses in each condition, each obtained from a separate pregnant dam. CP: cortical plate; SVZ: subventricular zone; VZ: ventricular zone; V: ventricular. Scale bars: 50 µm.

We previously showed that ethanol exposure resulted in decreased miR-153 expression in NSCs (Balaraman et al., 2012; Sathyan et al., 2007). Since Nfia and Nfib were direct targets of miR-153, we hypothesized that exposure to ethanol in an *in vivo* model would result in an increased expression of these factors. *In utero* ethanol exposure between GD12.5 and 14.5, i.e. during the first half of the second trimester equivalent period of cortical neurogenesis (Takahashi et al., 1995) results in an overall thinning of the cortical plate (Fig. 11a.i vs Fig. 11b.i) accompanied by ventriculomegaly as previously described (Sudheendran et al., 2013). While control fetuses expressed Nfia (Fig. 11a.i–a.iii) and Nfib (Fig. 11d.i–d.iii) mainly within the cortical plate, ethanol-exposed fetuses exhibited a broader expansion of Nfia (Fig. 11b.i,b.ii) and Nfib (Fig. 11e.i,e.ii) immunoreactivity throughout the extent of the dorsal telencephalic wall, including within the ventricular zone. At higher magnification (Fig. 11b.iii, Fig. 11e.iii) Nfia and Nfib immunoreactivity could be observed in mitotically active apical cells of the VZ in ethanol-exposed fetal brains.

### MiR-153 prevents and partly reverses effects of ethanol exposure on mRNA transcript levels

**Fig. 12.**
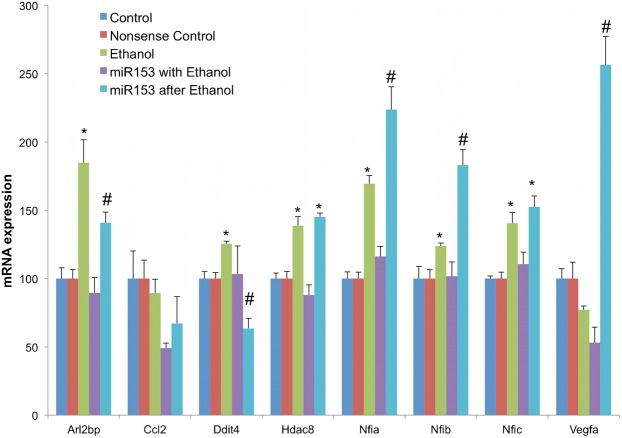
MiR-153 prevents and partly reverses ethanol's effects on miR-153-regulated gene transcripts. Bar graphs represent real-time RT-PCR analysis for mRNA expression of miR-153 sensitive genes in control neurosphere cultures (untreated or transfection control), ethanol (320 mg/dl) alone, miR-153 over-expression with ethanol exposure (prevention paradigm), or miR-153 over-expression for 48 hours after 5 days of ethanol exposure (reversal paradigm) of NSCs. The *y*-axis indicates normalized mRNA expression (normalized to 18s) relative to control samples. Data were expressed as mean±SEM. n = 4 independent experiments. *Significant difference from control. #Significant difference from ethanol-exposed. See Results section for p-values.

We next tested the prediction that because of its suppressive effect on miR-153, ethanol exposure would generally result in increased expression of miR-153-sensitive mRNA transcripts. We were also interested in examining the extent to which miR-153 could prevent or even reverse the effects of ethanol on NSCs. Neurosphere cultures were exposed to control medium or to ethanol, at a concentration (320 mg/dl) that was previously shown to suppress miR-153 (Balaraman et al., 2012; Sathyan et al., 2007), and was within the range of blood alcohol content achievable in alcoholics (Adachi et al., 1991). To assess the capacity of miR-153 to *prevent* ethanol's effects (prevention paradigm), some ethanol-treated cultures were concurrently exposed to miR-153 by transient transfection. To assess the capacity of miR-153 to *reverse* the effects of prior ethanol exposure (reversal paradigm), other ethanol-treated cultures were exposed to miR-153 for 48 hours following a five-day period of ethanol exposure. Our data (Fig. 12) showed a global (MANOVA_PTS_, F_(36,56)_ = 2.786, p<0.001), as well as a transcript-specific effect of treatment on mRNA expression (Nfia (ANOVA, F_(4,19)_ = 41.555, p<0.001), Nfib (F_(4,19)_ = 16.609, p<0.001), Nfic (F_(4,19)_ = 13.722, p<0.001), Ddit4 (F_(4,19)_ = 5.313, p<0.004), Hdac8 (F_(4,19)_ = 17.715, p<0.001), and Arl2bp (F_(4,19)_ = 14.9, p<0.001)). *Post-hoc* analyses showed that ethanol exposure did result in increased expression of Nfia (p<0.00003), Nfib (p<0.045), Nfic (p<0.0007), Arl2bp (p<0.0002), Ddit4 (p<0.044), and Hdac8 (p<0.0004), and that simultaneous over-expression of miR-153 prevented the inductive effect of ethanol on these mRNA transcripts (cultures administered miR-153 along with ethanol were not significantly different from controls). The increased expression of Nfia and Nfib mRNA following ethanol exposure validated the results from the *in vivo* exposure paradigm (Fig. 11) and indicate that ethanol directly influences Nfia/b expression in fetal neuroepithelial cells, rather than indirectly, *via* altered maternal–fetal physiology.

Over-expression of miR-153 *after* an episode of ethanol exposure (the reversal paradigm), on the other hand, resulted in varied outcomes. In the case of Ddit4 and Arl2bp, over-expression of miR-153 after ethanol exposure, resulted in the predicted reversal of transcript expression to control levels (p<0.0002 and p<0.027 relative to ethanol exposure, respectively). Nfic and Hdac8 mRNA expression was not reversed by subsequent miR-153 over-expression. However, over-expression of miR-153 after ethanol exposure resulted in a surprising and significant additional increase in Nfia, Nfib and Vegfa mRNA transcript levels relative to ethanol exposure alone (p<0.0004, p<0.0002, and p<0.1E−09, respectively). These data show that simultaneous exposure to miR-153 prevents ethanol induction of most miR-153 responsive transcripts, while sequential over-expression of miR-153 after ethanol exposure results in reversal of ethanol effects on some transcripts. However, sequential exposure to miR-153 following ethanol also unexpectedly resulted in an induction of transcripts (Nfia and Nfib) that are direct miR-153 targets.

### The partial nicotinic agonist, varenicline, induces expression of miR-153

**Fig. 13.**
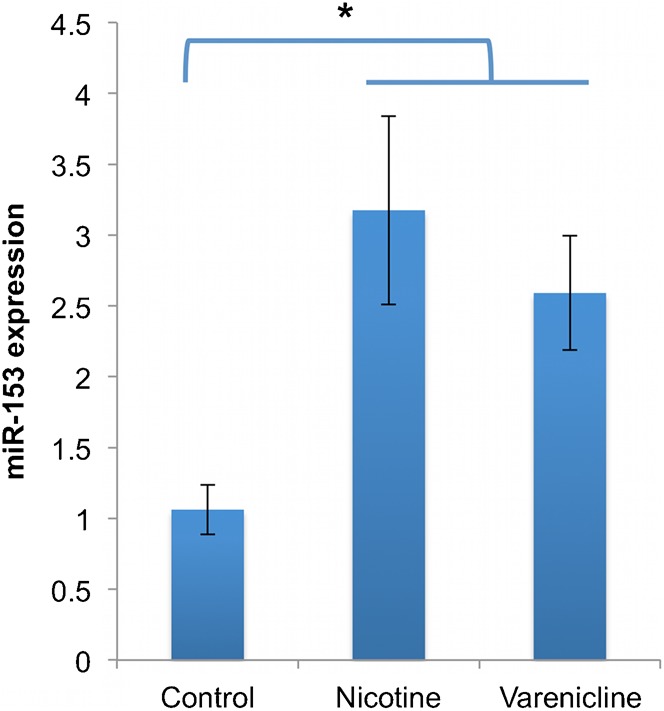
Nicotine and the nAChR partial agonist varenicline induce miR-153 expression. Bar graph depicts real-time RT-PCR expression of miR-153 in control, nicotine and varenicline-exposed neurosphere cultures. MiR-153 expression is significantly induced in nicotine and varenicline-treated groups. The *y*-axis indicates normalized miR-153 expression (normalized to U6) relative to control samples. Data were expressed as mean±SEM. n = 4 independent replicates. Asterisk indicates significant difference from control.

We previously reported that nicotine, acting at nAChRs, induced miR-153 expression and prevented the ethanol-mediated suppression of this miRNA in NSCs (Balaraman et al., 2012). We therefore re-tested the effect of nicotine on miR-153. Additionally, we tested the effect of varenicline, a partial nAChR agonist (Mihalak et al., 2006) and a Food and Drug Administration (FDA)-approved agent for smoking cessation (Hartmann-Boyce et al., 2013), because of recent evidence showing that it is also an effective treatment for alcohol use disorders (Litten et al., 2013; Steensland et al., 2007). Both nicotine and varenicline (each at 1.0 µM for 5 days) resulted in a statistically significant induction of miR-153 expression in neurosphere cultures (F_(2,15)_ = 5.621, p<0.015, Fig. 13). Consistent with the increase in miR-153 expression, *in utero* exposure to nicotine (1 mg/kg, twice a day) between GD12.5 and 14.5 resulted in a near complete loss of Nfia (white arrows, Fig. 11c.i,c.ii vs Fig. 11a.i,a.ii) and Nfib (white arrows, Fig. 11f.i,f.ii vs Fig. 11d.i,d.ii) immunofluorescence from the fetal cortical plate.

### Varenicline decreases expression of miR-153-dependent mRNAs and prevents and reverses effects of ethanol

**Fig. 14.**
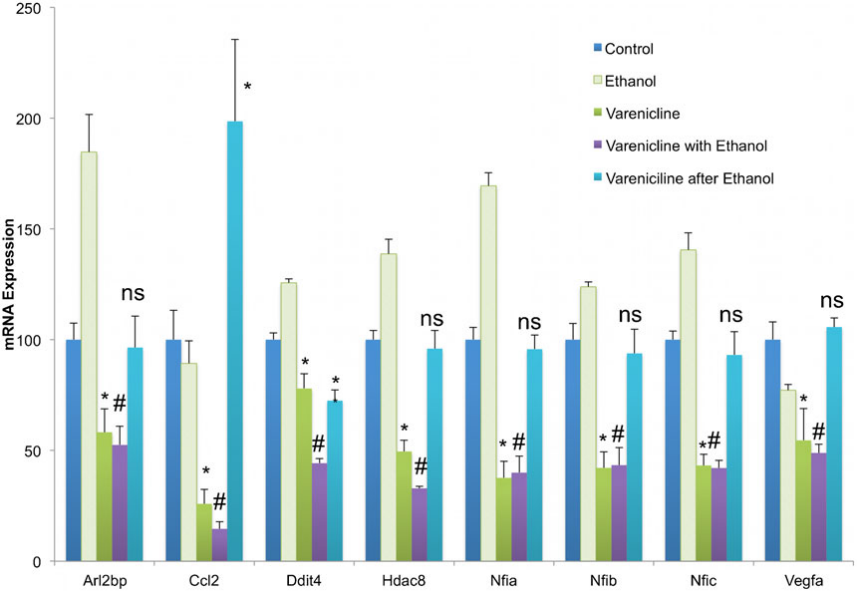
Varenicline prevents and reverses the effects of ethanol on miR-153 target gene expression. Bar graph depicts real-time RT-PCR analysis of mRNA expression of miR-153-regulated genes in control neurospheres, or neurospheres treated with varenicline (1 µM) alone, varenicline in combination with ethanol (prevention paradigm), and varenicline treatment for 48 hours following 5 days of ethanol exposure (reversal paradigm). The striped bars show reference ethanol exposure data from Fig. 9. The *y*-axis indicates normalized mRNA expression (normalized to 18s) relative to control samples. Data were expressed as mean±SEM. n = 4 independent replicates. *Significant difference from control. #Significant difference from ethanol-exposed. See Results section for p-values.

Based on the above data, we hypothesized that varenicline would prevent and reverse the effects of ethanol exposure on miR-153-regulated mRNAs. In the next series of experiments, we exposed neurosphere cultures to either control medium, to varenicline alone, to the ‘prevention paradigm’ (varenicline together with ethanol for 5 days), or to the ‘reversal paradigm’ (varenicline for 48 hours subsequent to a 5-day episode of ethanol exposure), and examined the regulation of miR-153 target gene transcripts. In all conditions, varenicline was administered at 1.0 µM, and ethanol at a concentration of 320 mg/dl. There was an overall significant effect (MANOVA_PTS_, F_(27,24)_ = 2.36, p<0.018), as well as a transcript-specific effect of nicotinic activation on miR-153-regulated transcripts (ANOVAs, F_(3,14)Nfia_ = 13.51, p<0.0002; F_(3,14)Nfib_ = 6.946, p<0.004; F_(3,14)Nfic_ = 16.1, p<8.15E−05; F_(3,14)Ddit4_ = 21.8, p<1.5E−05; F_(3,14)Hdac8_ = 21.18, p<1.8E−05; F_(3,14)Arl2bp_ = 3.55, p<0.04; F_(3,14)Vegfa_ = 5.031, p<0.014; and F_(3,14)Ccl2_ = 10.24, p<0.0008). Consistent with our finding that varenicline induced miR-153, we found that this partial nAChR agonist decreased expression of miR-153-target mRNA transcripts (*post hoc* fLSD relative to control, p_Nfia_<0.0002, p_Nfib_<0.003, p_Nfic_<0.0001, p_Ddit4_<0.008, p_Hdac8_<0.0002, p_Arl2bp_<0.037, p_Vegfa_<0.02, and p_Ccl2_<0.046, Fig. 14). Moreover, in the ‘prevention paradigm’ (the presence of varenicline), ethanol exposure did not result in increased mRNA transcript expression (*post hoc* fLSD relative to control, p_Nfia_<0.0003, p_Nfib_<0.003, p_Nfic_<9.82E−05, p_Ddit4_<1.8E−06, p_Hdac8_<9.14E−06, p_Arl2bp_<0.02, p_Vegfa_<0.01, p_Ccl2_<0.02, Fig. 14). Finally, in the ‘reversal paradigm’, varenicline exposure subsequent to ethanol exposure prevented the ethanol-induced increase in all miR-153 target transcripts (Nfia, Nfib, Nfic, Hdac8, Arl2bp, and Vegfa, all p = n.s. (not significantly different), or Ddit4, decreased, p<0.0008, relative to controls). Ccl2 represented an exception. Varenicline exposure (like miR-153) was unable to reverse the increase in Ccl2 expression due to previous ethanol exposure (p<0.006 relative to controls). These data show that varenicline mimics the effects of miR-153, and like miR-153, behaves as a functional antagonist to ethanol. Moreover, varenicline is able to both prevent and largely reverse the effects of ethanol exposure.

## DISCUSSION

Teratogens like ethanol are an established cause of birth defects. The fetal period of neurogenesis may constitute a particularly vulnerable period for brain development. Fetal NSCs produce a majority of neurons of the adult brain between the end of the first trimester through the second trimester-equivalent period (Bystron et al., 2008), before switching to gliogenesis during the third trimester-equivalent period (Miller and Gauthier, 2007). Therefore, alcohol exposure in the second trimester is likely to preferentially influence neurogenic programs. We previously observed in second trimester-equivalent *ex vivo* and *in vivo* models of neurogenesis that ethanol exposure did not result in NSC death, but instead interfered with NSC renewal and maturation (Camarillo and Miranda, 2008; Prock and Miranda, 2007; Santillano et al., 2005; Tingling et al., 2013). Ethanol-targeted miRNAs like miR-153 may mediate and explain some of ethanol's teratogenic effects. For example, ethanol's effects in neurosphere cultures were mimicked by loss of miRNAs, including miR-153 (Sathyan et al., 2007), and in a zebrafish model, developmental miR-153 knockdown was shown to mimic ethanol's effects on both craniofacial development and behavior (Tal et al., 2012). The question that arises is “Will identifying molecular targets of miR-153 provide us with insights into potential mechanisms for teratogenesis in NSCs?”.

Microarray and gene ontology analysis shows that miR-153 over-expression repressed cell-signaling pathways including GPCR pathways, which are important for NSC maturation (Callihan et al., 2011; Kobayashi et al., 2010). Collectively, repressed ontology categories were broadly associated with mature neural function, suggesting that miR-153 served as a repressor of neuronal differentiation in NSCs. These implicated ontology categories are consistent with our observations that miR-153-over-expressing cells exhibit deficient morphological transformation when cultured in a mitogen-withdrawal differentiation paradigm. We also observed that miR-153 over-expression *in vivo* within the SVZ coincided with loss of DCX immuno-labeling providing further evidence that miR-153 is a repressor of neuronal differentiation. We next sought to determine if miR-153 interfered with neuronal lineage commitment by examining the expression of CD24, because previous observations indicated that CD24+ precursors are committed to neuronal lineage (Nieoullon et al., 2005; Pastrana et al., 2009; Pruszak et al., 2009; Tingling et al., 2013; Yuan et al., 2011). However, whereas ethanol exposure resulted in a loss of CD24+ cells both *in vivo* and in cell culture (Tingling et al., 2013), miR-153 over-expression did not result in loss of CD24 expression, suggesting that miR-153, unlike ethanol, does not interfere with neuronal lineage commitment of VZ and SVZ precursors, but like ethanol, does interfere with subsequent neuronal differentiation. MiR-153 over-expression also did not alter cell survival, and unlike ethanol (Santillano et al., 2005), did not alter cell proliferation. These data suggest that loss of miR-153 may explain some, but not all, ethanol effects on NSCs. Moreover, these data are collectively consistent with recent evidence that miR-153 prevents the development and maturation of motor neurons (Wei et al., 2013), and serves as a translational repressor for synaptic and signaling proteins that are important for neuronal maturation and function (Doxakis, 2010; Long et al., 2012; Wei et al., 2013). While current analysis focused on miR-153-repressed transcripts, potential direct effects on induced transcripts cannot be discounted. MiRNA interactions with promoter regions of mRNA coding genes reportedly result in increased gene transcription (Place et al., 2008), and presumptive binding sites on lncRNAs serve as a mechanism for regulating miRNA function (Hansen et al., 2013). Therefore, potential interactions of miR-153 with induced mRNAs and lncRNAs need further assessment. Conversely, repressed mRNA transcripts like ARL2BP, CCL2 and VEGFA, which did not contain predicted miR-153 binding sites within their 3′UTRs, may be indirect miRNA targets. Their connection with miR-153 also needs further assessment.

The effects of miR-153 are likely to be mediated by a large network of both directly and indirectly regulated genes. This network includes the nuclear factor-1 family, Nfia, Nfib, and Nfic, which were all suppressed by miR-153 in neurosphere cultures. Moreover, *in utero* over-expression of pre-miR-153 coincided with loss of nuclear Nfia/Nfib immunofluorescence, and miR-153 over-expression in the ventricular zone (VZ) resulted in disrupted Nfia/Nfib expression in the overlying cortical plate, suggesting that miR-153 regulates the Nf1 family in the developing brain. Our data collectively show that members of the Nf1 family are specific, direct targets of miR-153. Both Nfia and Nfib possess extended 3′UTRs, which are predicted to assume complex, branched stem–loop structures, and are likely, as with other long 3′UTRs (Irier et al., 2009), to serve as a focus for regulated, context-dependent translation. In each of these transcripts, experimental analysis showed the presence of specific, active miR-153 binding sites that, based on the predicted folding of the 3′UTRs, are in close proximity to both 5′ and 3′-termini of the 3′UTRs, positioning these sites to influence translation. Interestingly, other equally strong predicted 3′UTR target sites were found to be non-functional, perhaps because they are masked in NSCs. However, a role for these cryptic sites at other stages of neuronal differentiation cannot be discounted.

Since developmental exposure to ethanol suppressed miR-153 expression, we predicted that *in utero* ethanol exposure would result in increased expression of Nfia and Nfib. Our data from both *in vivo* and neurosphere models show that this is indeed the case. *In vivo*, in control animals, at GD15, Nfia and Nfib expression were mainly localized to the emerging cortical plate consistent with the published literature (Plachez et al., 2008). However, following episodes of ethanol exposure, Nfia and Nfib-like immunofluorescence could be observed throughout the sub-ventricular and ventricular zones in addition to the cortical plate. This dysregulated pattern of expression is certainly interpretable as indicating general exposure-induced delay in fetal development. However, these data were validated in the neurosphere culture model where ethanol exposure resulted in increased expression of Nf1 family transcripts, supporting a direct relationship between exposure and target gene expression.

While little is known about miR-153, an extensive literature documents the developmental role of the Nf1 family. During the neurogenesis period, Nfia and Nfib are dynamically expressed in differentiating cortical plate neurons (Plachez et al., 2008). Nfia expression in neuronal lineage-committed precursors and in young neurons promotes their neural differentiation, but decreases self-renewal capacity of the remaining NSCs and causes them to switch from neurogenesis to gliogenesis (Barry et al., 2008; Deneen et al., 2006; Namihira et al., 2009; Piper et al., 2010; Wang et al., 2010). Ethanol is a complex teratogen, and its effects are likely to be broader than those mediated by a single miRNA and its target mRNA transcripts. However, since the Nf1 family serves as a feedback inhibitor for NSC self-renewal (Piper et al., 2010), these data advance an explanation for the observed loss of NSCs (Santillano et al., 2005; Tingling et al., 2013), following ethanol exposure. This hypothesized linkage between miR-153, the Nfia family and fetal alcohol neurodevelopmental defects needs further assessment especially in light of associations between Nfia haplo-insufficiency and brain malformations in human populations (Lu et al., 2007). It is possible that both over and under-expression of the Nfia family results in neurodevelopmental defects. Interestingly, bioinformatics analysis (http://inia.icmb.utexas.edu) indicates that members of the Nfia family are also up-regulated in human (Ponomarev et al., 2012) and animal (Mulligan et al., 2006) models of adult alcoholism. These transcription factors may constitute a component of both developmental and adult neural responses to alcohol exposure.

An important question is “Can we progress beyond the diagnosis of teratology towards developing intervention strategies to prevent or even reverse fetal growth defects?”. Education strategies aimed at limiting maternal alcohol consumption have been modestly successful at best, since the incidence of FAS in the United States has remained constant (0.05–0.2% of live births (Bertrand et al., 2005)) over the 40-year period since the syndrome was first described, while in countries like South Africa the prevalence rate is reportedly as high as 9% (May et al., 2013). Therefore alternate approaches, including biomedical interventional approaches designed to directly influence fetal development, need further investigation. In this context, the published literature implicating miRNAs like miR-153 as mediators of teratogenesis (Sathyan et al., 2007; Tal et al., 2012) is important, because miRNAs can be manipulated to promote neuro-protection *after* the onset of neuro-trauma (Selvamani et al., 2012). We therefore asked whether simultaneous over-expression of miR-153 *prevented*, and sequential over-expression *reversed*, ethanol's effects on target genes. Our data indicate that simultaneous over-expression of miR-153 effectively prevents ethanol-mediated induction of miR-153 targets. Moreover, miR-153 over-expression following ethanol exposure did reverse the effect of ethanol on some genes, i.e. Arl2bp and Ddit4. Surprisingly, and contrary to our prediction, miR-153 over-expression following ethanol exposure resulted in significantly increased transcript levels of Nfia and Nfib, even though these transcripts are direct targets of miR-153. The mechanism for this up-regulation is unknown. It is possible that the post-ethanol inductive effect of miR-153 on Nfia and Nfib is indirect, i.e. due to repression of an intermediate regulatory factor. However, collectively, these data provide the first promising evidence that miRNA manipulation successfully prevents, and even partially reverses a teratogen's effects on NSCs.

We next assessed the possibility that pharmacological interventions could prevent the inductive effects of ethanol on miR-153 target genes. We replicated our previously published observations (Balaraman et al., 2012) that low-dose nicotine exposure (at 1 µM) resulted in increased miR-153, and that as would be predicted, nicotine exposure *in vivo*, albeit at a relatively high dose, resulted in loss of expression of miR-153's direct targets, Nfia and Nfib, in the fetal cortical plate. Moreover, low-dose exposure to the partial nAChR agonist, varenicline also induced miR-153 expression. While varenicline, like nicotine may well regulate the expression of multiple miRNAs, exposure to varenicline along with ethanol in the *ex vivo* neurosphere model did prevent the ethanol induction of miR-153 target mRNAs. Importantly, varenicline administration *subsequent to* ethanol exposure, reversed the ethanol-induction of nearly all of the assessed miR-153-regulated transcripts, including importantly, Nfia/b/c. Varenicline is not only a clinically effective, FDA-approved, smoking-cessation agent (Hartmann-Boyce et al., 2013), but has been found to be effective in reducing alcohol craving in preclinical (Steensland et al., 2007) and human studies (Litten et al., 2013). Nicotinic receptor-mediated pharmacotherapies entail complex considerations of dose, timing and route of delivery (Matta et al., 2007), which are beyond the scope of the current study. However, these data, as well as the *in vivo* effects of nicotine exposure, resulting in suppression of neural Nfia/b expression, provide a rationale for further analysis of the therapeutic potential of varenicline, an agent that provides benefit to the mother by mitigating drug-seeking behavior, and also effectively mimics miR-153 in preventing and reversing the effect of a teratogen on fetal NSCs.

### Conclusions

**Fig. 15.**
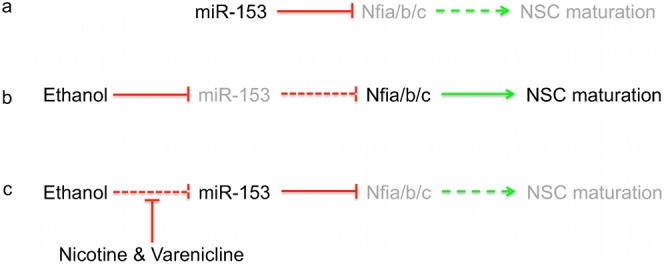
Model for the hypothesis that a network of miR-153 and the NF1 neurogenic transcription factor family (Nfia/b/c) mediate the effects of ethanol on NSC maturation. (a) miR-153 is a direct and negative regulator of NF1 expression. Our data on miR-153 regulation of gene expression and published data on NF-1 suggest that suppressing NF1 will in turn retard NSC maturation, and be predicted to be permissive of continued NSC renewal. (b) Ethanol suppresses miR-153, resulting in release of NF1 expression, potentially explaining findings that ethanol promotes NSC maturation. (c) Nicotinic (nAChR) activation prevents ethanol suppression of miR-153 and may serve to mitigate premature NSC differentiation. Green arrows depict positive regulation while red bars indicate negative regulation. Dashed lines indicate diminished regulation, while gray text indicates diminished expression or function.

Here, we identified a novel and direct inhibitory connection between a teratogen-targeted miRNA and the neurogenic Nf1 transcription factor family. These data predict an intriguing and testable hypothesis, i.e. that loss of miRNA control over neurogenic transcription factors may result in premature NSC maturation (Fig. 15), perhaps leading to loss renewal capacity, premature closure of the neurogenic window, and impaired brain development. Importantly, these data also show, in pre-clinical models, the translational potential for miRNAs, as a means to therapeutically intervene in fetal development. Pharmacological approaches to miRNA manipulation, particularly those involving nicotinic receptor activation, are certainly controversial in light of the long-documented teratogenic effects of fetal nicotine exposure (Nishimura and Nakai, 1958). However, successful pharmacological interventions to reverse developmental defects are likely to entail using agents that are themselves teratogenic. Precedence for using a teratogen to mitigate developmental defects comes from data showing that the alkaloid teratogen and hedgehog pathway antagonist, cyclopamine, which causes craniofacial defects in fetal mice (Lipinski et al., 2008), was nevertheless able, at sub-teratogenic doses, to partly rescue craniofacial defects due to genetic ablation of the transcription factor, SP8 (Kasberg et al., 2013). With careful attention to factors like dose and timing of exposure, *in vivo* pharmacological manipulations of miRNAs and their target gene networks may well be a feasible approach to prevent and perhaps even reverse fetal damage following teratogen exposure.

## MATERIALS AND METHODS

### Isolation and expansion of mouse cortical neural precursors

Timed pregnant mice (C57Bl6-SJ, Harlan Laboratories) were housed in AAALAC accredited facilities at Texas A&M Health Science Center. All animal procedures were approved by the University Laboratory Animal Care Committee. Cortical neuroepithelial tissues were isolated from the dorsal telencephalic vesicles, corresponding to the region of the future isocortex, of gestational day (GD) 12.5 mouse fetuses (GD0 was defined as the day the dams were sperm-positive), and neurosphere cultures were established according to previously published protocols (Camarillo et al., 2007; Prock and Miranda, 2007; Santillano et al., 2005). Briefly, approximately 1∼2 million neural precursor cells isolated from the cortical region from fetuses of both sexes were combined and cultured in serum-free mitogenic medium DMEM/F12 (number 11330-032; Life Technologies, CA), 20 ng/ml bFGF (basic fibroblast growth factor; number 354060, BD Biosciences, CA), 20 ng/ml hEGF (human epidermal growth factor; number 53003-018, Life Technologies, CA), 1% ITS-X (insulin–transferrin–selenium–X; number 51500-056, Life Technologies, CA), 0.15 ng/ml LIF (leukemia inhibitory factor; number L200, Alomone Labs), 0.85 U/ml heparin (number 15077-019, Life Technologies, CA), and 20 nM progesterone (number P6149, Sigma, MO). Neurospheres were mechanically dissociated into single cells with medium changed every two or three days. These cultures maintain stem cell markers and renewal characters (Santillano et al., 2005; Tingling et al., 2013). To assess the differentiation capacity of control or miR-153 over-expressing NSC populations, transfected neurospheres were cultured on laminin-coated (0.5 mg/ml) glass coverslips (number 16004-342; VWR, PA) in 6-well plates for 48 hours, in a mitogen-withdrawal paradigm (+bFGF/−EGF/−LIF) that results in stereotypic transformation of NSCs into early migratory bipolar cells and expression of neuronal markers (Camarillo et al., 2007; Camarillo and Miranda, 2008). Cultures were fixed in ice-cold methanol for immunofluorescence analysis.

### Neurosphere culture treatment paradigm

#### Ethanol exposure

Neurosphere cultures containing ∼3 million cells were assigned to either a vehicle control, or an ethanol-treated group (320 mg/dl, 70 mM) for 5 days, to bracket a period equivalent to the peak neurogenic period in the mouse. The ethanol concentration in culture medium was determined by an alcohol analyzer (Analox instruments, MA). The particular dose of ethanol was chosen to reflect levels within ranges attainable during episodes in chronic alcoholics (Adachi et al., 1991), and is expected to reflect levels attained by a fetus following prenatal exposure (Gottesfeld et al., 1990). Four independent samples from either control or ethanol-treated groups were collected for further analysis.

#### Varenicline exposure

Varenicline tartrate (Tocris Bioscience, Bristol, UK) at 1.0 µm was administered alone or in combination with 320 mg/dl ethanol according to previous protocols established in our laboratory (Balaraman et al., 2012), and four independent samples from each group were collected for analysis.

#### MiR-153 mimetic exposure

The human genome encodes two copies of miR-153 (mir-153-1 and miR-153-2), whereas the mouse genome encodes a single copy of this miRNA (Mandemakers et al., 2013). The reported experiments utilized mouse (*mmu*)-miR-153 as an experimental model. Furthermore, we focused on a strategy of miR-153 over-expression rather than repression, as a means to prevent and reverse ethanol's effects on NSCs. Neural progenitors were exposed to *mmu*-miR-153 mimetic (miRNASelect™ pEGP-mmu-miR-153 Expression Vector, Cell Biolabs, CA) or control (miRNASelect™ pEGP-miR Null Control Vector, Cell Biolabs, CA), either alone or in combination with ethanol at 320 mg/dl. The miRNA expression construct also encoded a green fluorescent protein (GFP)/puromycin resistance fusion protein (Fig. 1a) for monitoring transfection efficiency and as a selection marker. For cell transfection experiments, neurospheres were collected and trypsinized into a single cell suspension. Cell density was determined by a Countess® Automated Cell Counter (Life Technologies, CA). A 10 µl cell suspension containing 3∼4 million cells was transfected by electroporation with 7 µg of either control or miR-153 precursor clone using a NEON electroporator with transfection kit (settings 1200 V, 20 ms, 2 pulses, Life Technologies, CA). Independent replicate experiments were harvested for further analysis.

### MiRNA and mRNA isolation, mouse whole genome microarray analysis

MiRNA and mRNA were extracted from harvested cells using a *mir*Vana™ miRNA Isolation Kit (number AM1560; Life Technologies, CA) according to the manufacturer's protocol. Purified miRNA and mRNA samples were quantified by NanoDrop® ND-1000 UV-Vis Spectrophotometer (Thermo Scientific, MA). For mRNA microarray analysis, 1.0 µg of purified mRNA samples from either control or miR-153 mimetic-treated groups, mixed with 50 pg RNA spike-in control, were used to generate biotin labeled cRNA samples by a linear amplification method using Ambion's MessageAmp™ II-Biotin Enhanced Single Round aRNA Amplification Kits (number Am1791; Life Technologies, CA). 10 µg labeled cRNA samples were fragmented at 94°C for 20 minutes, mixed with hybridization buffer (Applied Microarray, Tempe, AZ) and hybridized with CodeLink whole genome arrays (Applied Microarray). Post-hybridization processing and secondary-labeling with Cy™5-conjugated to Streptavidin were performed according to the manufacturer's instructions (Applied Microarray). Microarrays were scanned using a GenePix 4000B scanner (Molecular Devices, Sunnyvale, CA). A total of 12 microarrays (six in each condition) were used to assess changes in the transcriptome. Array images were processed using CodeLink™ software (Applied Microarray, AZ), and global median normalization was used to generate normalized expression values. The data were analyzed further, using GeneSifter Analysis Edition (GSAE, PerkinElmer-Geospiza, Seattle, WA).

### Real-time RT-PCR validation for miRNA and mRNA

MiRNA expression of control and miR-153 mimetic-treated samples was validated before performing mouse whole genome microarray analysis. Briefly, 25 ng of purified total RNA was used to generate cDNA, using a Universal cDNA Synthesis Kit according to the manufacturer's protocol (number 203300; Exiqon, Denmark). cDNA samples were diluted 80× and 4 µl was used as a PCR reaction template in a 10 µl PCR reaction. PCR reactions were run in triplicate on an Applied Biosystems 7900HT real-time PCR instrument (Applied Biosystems, CA) using a SYBR green-based real-time PCR reaction kit (number 203450; Exiqon, Denmark) with miR-153 (number 204338; Exiqon, Denmark) and U6snRNA primer sets (number 203907; Exiqon, Vedbaek, Denmark). U6snRNA was used as a normalization control. Real-time RT-PCR data of miRNA expression were quantified using the SDS 2.4 software package (Applied Biosystems, CA). Transfection with the miR-153 mimetic resulted in a ∼30-fold induction of miR-153 expression relative to control groups, without a statistically significant change in another ethanol-sensitive miRNA, miR-21 (data not shown). RT-PCR of mRNA was performed to evaluate expression of candidate miR-153 target transcripts that were identified in the mouse whole genome microarray analysis. Total RNA (500 ng) was used to generate first strand cDNA using qScript cDNA Supermix kit (number 95048-100; Quanta Biosciences, MD). Real-time PCR was performed on a 7900HT Real-Time PCR System (Life Technologies, CA) using the PerfeCTa SYBR Green SuperMix with ROX kit (number 95053-500; Quanta Biosciences, Gaithersburg, MD). 2.0 µl of cDNA was used as the template in a reaction volume of 10 µl. RNA expression was quantified by calculating the difference between the cycle threshold of the mRNA of interest, and the reference gene (18s mRNA) for each sample. Specificity of the amplification was evaluated by thermal stability analysis of the amplicon. Individual and reference gene primer sets (Integrated DNA Technologies, Coralville, Iowa) are listed in Table 4.

**Table 4.**
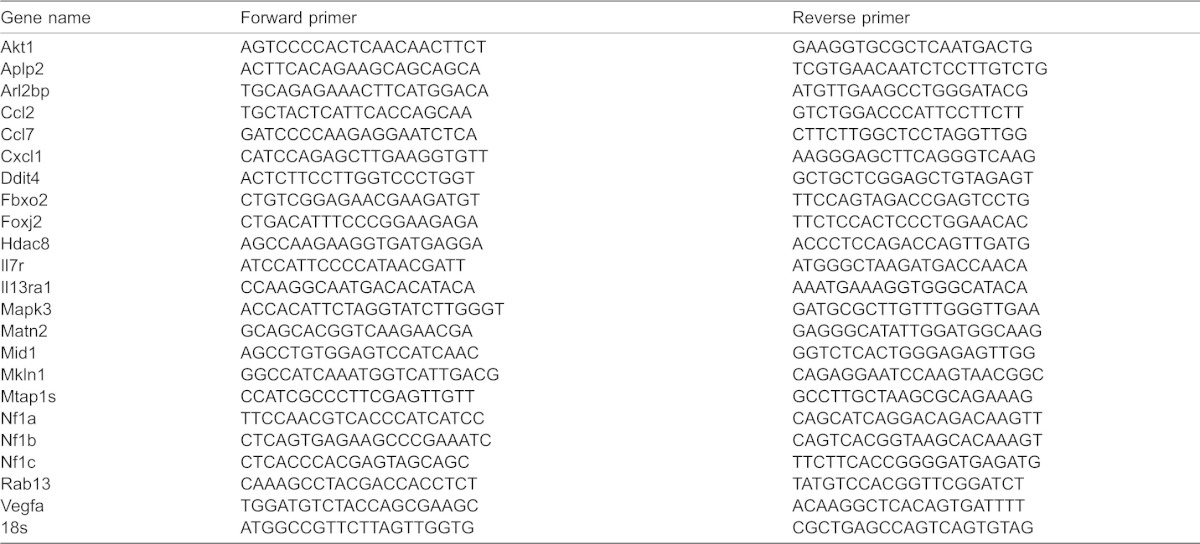
List of real time RT-PCR primer sequences

### 3′UTR analysis of miR-153 candidate genes

Plasmids containing 3′UTR firefly/Renilla Duo-Luciferase reporter luciferase constructs (miTarget™ 3′UTR miRNA Targets, GeneCopoeia, Rockville, MD) were amplified and purified using a EndoFree plasmid Maxi kit (number 12362; Qiagen, Germantown, MD) according to manufacturer's protocol. Purified plasmids were quantified using a NanoDrop® ND-1000 UV-Vis Spectrophotometer (Thermo Scientific, Waltham, MA). 150 ng of 3′UTR construct (Matn2 (number MmiT031425; GeneCopoeia), Vegfa (number MmiT024368; GeneCopoeia), Nfia (number MmiT054793; GeneCopoeia), Nfib (number MmiT027729; GeneCopoeia), pmiR153-Luc (a positive control for miR-153 translation repression activity, number LR-0064; Signosis, Sunnyvale, CA)), 100 ng of either control or miR-153 mimetic expression plasmids (Cell Biolabs, CA), and 10 µm of control or targeted morpholino antisense, anti-3′UTR oligonucleotides (Gene Tools, Philomath, OR; to protect predicted miR-153 binding sites in 3′UTRs of target mRNA transcripts), were co-transfected into 2×10^4^ cells, using a NEON electroporator according to manufacturer's protocol (as outlined above). At 24 hours after transfection, luciferase signals were determined by Dual-Glo® luciferase assay (number E2920; Promega, Madison, WI), and quantified using the Synergy-2 multi-mode plate reader (BioTek, Winooski, VT). Firefly luciferase intensity from each sample was normalized to the internal control, Renilla luciferase.

### Ultrasound-guided *in utero* electroporation

Ultrasound guided micro-injection procedures were adapted from our previously published protocol (Tingling et al., 2013). GD13.5 timed pregnant mice were anesthetized with 4% isoflurane and 0.5% oxygen, then positioned on a heated mouse platform (Integrated Rail System, VisualSonics, Toronto, Canada) to monitor temperature, respiration, and heart rate. Maternal core body temperature was maintained at 33–35°C and maternal heart rate was monitored with adjustments to the level of anesthesia made to maintain a constant heart rate of ∼450 beats/minute. Following hair removal (Nair® hair remover), the skin was sterilized with ethanol (80% v/v) followed by Betadine®. A midline incision was made through the skin and peritoneal wall, and one uterine horn was gently exteriorized through the incision, and carefully drawn through a Parafilm® flap in the bottom of a sterilized Petri dish. The uterine horn was completely immersed in warmed ultrasound gel (Ecogel, Eco-Med, Mississauga, Canada). The brain and lateral ventricle of each fetus was located by ultrasound imaging (VEVO2100, VisualSonics, Toronto, Canada). Once the fetal orientation was determined, 5 µg of control or miR-153 plasmid was injected with a micropipette, under ultrasound guidance, through the uterine wall, into the fetal lateral ventricle. Fetuses of either sex were used for microinjection experiments. A forceps-type electrode connected to a BTX ECM 630 electroporator (4 pulses, each at 35 V and 950 msec duration, Harvard Apparatus, Holliston, MA) was used to electroporate the plasmid into the fetal telencephalon. Following electroporation, the uterine horn was reinserted into the peritoneal cavity and the abdominal wall and skin were closed with sutures. Mice were sacrificed and the injected embryos were collected after 48 hours post-transfection. Collected embryos were fixed in 4% buffered paraformaldehyde, cyro-protected with 30% sucrose, and cryo-sectioned at 20 µm.

### *In utero* ethanol and nicotine exposure models

Timed pregnant mice were exposed to ethanol (3 gm/kg) or an equivalent volume of water by intragastric gavage twice daily between GD12.5 and 14.5, bracketing the first half of the second trimester-equivalent period of cerebral cortical neurogenesis (Takahashi et al., 1995), according to our previously published protocols (Bake et al., 2012; Sudheendran et al., 2013; Tingling et al., 2013). The dose of ethanol results in a peak blood alcohol content of 117 mg/dl (Bake et al., 2012) that can be attained in humans following binge episodes of ethanol consumption. A second group of animals were exposed to intra-peritoneal (i.p.) injections of saline (controls) or nicotine (1 mg/kg free-base, Sigma (N3876); in saline) twice daily between GD12.5 and 14.5 as outlined above. The dose, approximating the nicotine content of a cigarette, and route of exposure were selected based on published literature for mouse studies, and was twice that of the effective dose for behavioral alterations but less than 1/5th of the effective dose required for seizure induction (Matta et al., 2007). At the end of the exposure period, one fetus from each dam (four each of gavage and i.p. controls and four ethanol- and nicotine-exposed in total of either sex) was cryo-sectioned and subjected to immunofluorescence analysis.

### Immunofluorescence analysis

Mouse tissue sections and cell cultures were incubated in a blocking solution (10% normal serum, 0.6% Triton X-100 in PBS) at room temperature for 1 hour. Tissues were incubated with primary antibody overnight at 4°C in appropriate dilution (anti-GFP to visualize co-expression of miR-153 (1:800; number ab13970; Abcam, MA), anti-Nfib (1:100, number HPA003956; Sigma–Aldrich, MO), anti-Nfia (1:100, number AP14133b, Abgent, CA), anti-Hdac8 (1:400; number ab39664, Abcam, MA), anti-DCX (1:800; number ab18723, Abcam, MA) and anti-CD24 (1:300; number ab64065, Abcam, MA) and MAP2 a&b (Ab5622, 1:300; Millipore). Slides were washed in PBS three times, and Alexa-Fluor conjugated secondary antibodies were used in order to visualize. (numbers A11001, A11032, A11037; Life Technologies, CA; numbers SC-362261, SC-362271; Santa Cruz Biotechnology, CA). Cultured cells were also immuno-labeled with ant-GFP antibody. Cells and tissues were mounted with fluorescence mounting medium containing DAPI (number H-1200; Vector Laboratories, CA) and photographed using an Olympus microscope.

### Cell proliferation assay

DNA synthesis was measured using the Click-iT® EdU cell proliferation assay (Life Technologies, C-10352) according to our published protocol (Tingling et al., 2013). Briefly, 4×10^5^ cells were treated with 1.5 µg of the scrambled control-GFP or pre-miR-153-GFP expression vector followed by incubation with 10 µM EdU (5-ethynyl-2′-deoxyuridine) for 16 hours to monitor DNA synthesis. Cells were fixed, washed, and Incorporated EdU was detected by covalently binding Alexa Fluor® 555-azide. The percent of EdU-labeled cells was then quantified with an Olympus fluorescence microscope.

### Apoptosis assay

Apoptosis was detected by a Homogeneous Caspase Assay (number 03005372001, Roche Applied Science, IN). Briefly, cell aliquots (4×10^5^ cells) were treated with 1.5 µg of the scrambled control-GFP or pre-miR-153-GFP expression vector for 12 hours. Staurosporine (2 µM for 2 hours)-treated neurosphere cultures and U937 cells treated with camptothecin (4 µg/ml for 4 hours) were used as positive controls. Caspase activity was measured by adding the diluted caspase substrate (DEVD-R110) at 37°C for 2 hours. Cleavage of the caspase substrate and release of the fluorescent dye, Rhodamine 110, was determined at λ_max_ = 521 nm with the fluorescence microplate reader (Tecan Infinite M200, Switzerland).

### Data analyses

Sample sizes from each group ranged from 4 to 6 independent replicates. Sholl analysis of neurite length was conducted using ImageJ. The total number of intersections was counted for each cell, as a measure of neurite extension. Median-normalized mRNA microarray data were analyzed by T-tests with Benjamin and Hochberg correction for multiple comparisons, using GeneSifter Analysis Edition (GSEA, Perkin-Elmer/Geospiza, WA), with a fold-change threshold set at ±1.3-fold. Gene ontology analysis was conducted and z-scores computed for over-representation analysis. Data from all other experiments were analyzed by multivariate (Pillai's trace statistic, MANOVA_PTS_) or univariate (ANOVA) analysis of variance. MANOVAs were followed by *post hoc* univariate ANOVA, and planned comparisons were further computed with the Fisher's least significant difference (f-LSD) test (SPSS v20, IBM). Single comparison experiments were evaluated with T-tests with the statistical significance set at p<0.05. Data are expressed as mean±SEM.
